# Lung cancer intravasation-on-a-chip: Visualization and machine learning-assisted automatic quantification

**DOI:** 10.1016/j.bioactmat.2025.06.028

**Published:** 2025-06-27

**Authors:** Christy Wing Tung Wong, Joyce Zhi Xuen Lee, Anna Jaeschke, Sammi Sze Ying Ng, Kwok Keung Lit, Ho-Ying Wan, Caroline Kniebs, Dai Fei Elmer Ker, Rocky S. Tuan, Anna Blocki

**Affiliations:** a Institute for Tissue Engineering and Regenerative Medicine, The Chinese University of Hong Kong, Hong Kong SAR, China; b School of Biomedical Sciences, Faculty of Medicine, The Chinese University of Hong Kong, Hong Kong SAR, China; c Center for Neuromusculoskeletal Restorative Medicine (CNRM), Hong Kong Science Park, Shatin, New Territories, Hong Kong SAR, China; d Department of Biohybrid and Medical Textiles (BioTex), AME - Institute of Applied Medical Engineering, Helmholtz Institute, RWTH Aachen University, Aachen, Germany; e Department of Orthopaedics & Traumatology, Faculty of Medicine, The Chinese University of Hong Kong, Hong Kong SAR, China; f Department of Biomedical Engineering, The Hong Kong Polytechnic University, Hong Kong SAR, China

**Keywords:** Cancer intravasation, Lung cancer, Epithelial-to-mesenchymal transition (EMT), Macrophages, Transforming growth factor-beta 1 (TGF-β1), Microfluidic devices, Machine learning-assisted image processing, Image segmentation, Pattern recognition, Random forest

## Abstract

During lung cancer metastasis, tumor cells undergo epithelial-to-mesenchymal transition (EMT), enabling them to intravasate through the vascular barrier and enter the circulation before colonizing secondary sites. Here, a human *in vitro* microphysiological model of EMT-driven lung cancer intravasation-on-a-chip was developed and coupled with machine learning (ML)-assisted automatic identification and quantification of intravasation events.

A robust EMT-inducing cocktail (EMT-IC) was formulated by augmenting macrophage-conditioned medium with transforming growth factor-β1. When introduced into microvascular networks (MVNs) in microfluidic devices, EMT-IC did not affect MVN stability and physiologically relevant barrier functions.

To model lung cancer intravasation on-a-chip, EMT-IC was supplemented into co-cultures of lung tumor micromasses and MVNs. Wihin 24 h of exposure, EMT-IC facilitated the insertion of membrane protrusions of migratory A549 cells into microvascular structures, followed by successful intravasation. EMT-IC reduced key basement membrane and vascular junction proteins - laminin and VE-Cadherin - rendering vessel walls more permissive to intravasating cells. ML-assisted vessel segmentation combined with co-localization analysis to detect intravasation events confirmed that EMT induction significantly increased the number of intravasation events.

Introducing metastatic (NCI-H1975) and non-metastatic (BEAS-2B) cell lines demonstrated that both, baseline intravasation potential and responsiveness to EMT-IC, are reflected in the metastatic predisposition of lung cancer cell lines, highlighting the model's universal applicability and cell-specific sensitivity.

The reproducible detection of intravasation events in the established model provides a physiologically relevant platform to study processes of cancer metastasis with high spatio-temporal resolution and short timeframe. This approach holds promise for improved drug development and informed personalized patient treatment plans.

## Highlights

• The human cancer intravasation-on-a-chip features cancer spheroids and microvascular networks.
• Robust EMT is induced by macrophage-conditioned medium augmented with TGF-β1.
• Upon EMT facilitates the insertion of membrane protrusions and cancer intravasation.
• Intravasation events were detected by machine learning-assisted quantification.

## Graphical abstract

**Figure:**
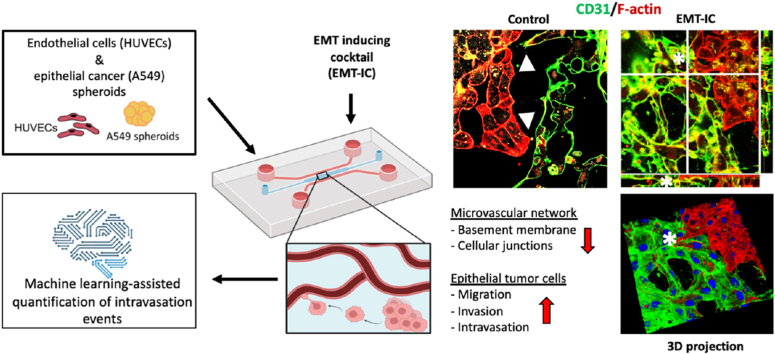

## 1 Introduction

Lung cancer is the leading cause of cancer-related deaths worldwide [1]. It is often only diagnosed at advanced metastatic stages when treatment options are limited, resulting in 5-year relative survival rates as low as only 9 % [2]. It is thus of high importance to develop physiologically relevant models of human metastatic lung cancer to enable a better study of the underlying pathological processes, identify suitable drug targets, and for high throughput drug screening. Microphysiological models of cancer metastasis have the potential to bridge the gap between 2D cell culture models, animal models and the actual clinical situation, thereby addressing the high attrition rates in drug development and to fulfill the transition toward personalized medicine [3].

Lung tumors can be divided into non-small cell lung cancer (NSCLC), comprising around 80–85 % of all lung cancer cases, and small cell lung cancer (SCLC), comprising the other 15 % of the cases [4]. For epithelial lung cancer to metastasize, cancer cells from the primary solid tumor usually undergo five key stages, namely local invasion driven by a hallmark process called epithelial to mesenchymal transition (EMT), intravasation, dissemination/circulation, extravasation, and finally colonization as a secondary tumor [5]. Indeed, various microphysiological systems of cancer metastasis exist. They mainly focus on later stages of cancer metastasis, such as extravasation events [[6], [7], [8], [9], [10]]. It is important to note though that among the five stages, EMT and intravasation are the rate limiting steps, determining the number of circulating tumor cells with the potential to metastasize [11]. Tackling EMT, local invasion and intravasation could in principle minimize and/or prevent the systemic spread of cancer cells at its start and in this way improve the clinical outcome of lung cancer patients. There is thus a particular need to establish human microphysiological models of EMT-driven cancer intravasation.

Indeed, cancer intravasation has been heavily studied using a range of *in vivo* [[12], [13], [14], [15]] and *in vitro* models. *In vivo* models utilize chick embryos, fluorescent transgenic zebrafish, and murine models. They assess cancer cell intravasation indirectly by measuring the number of circulating tumor cells or directly by utilizing non-invasive, high-resolution imaging techniques [[13], [14], [16]]. Although these models enable the study of tumor progression in a complex physiological environment, *in vivo* animal models of cancer intravasation have low animal-to-human transitional rates from preclinical to clinical treatment, resulting in increasing concerns regarding the use of animals as predictive tools for human responses [[13], [15], [17]]. These issues raised the need for physiologically relevant human *in vitro* models to investigate cancer biology and therapeutic development.

Currently, *in vitro* models include Transwell migration/invasion assays, where a porous membrane seeded with a 2D layer of endothelial cells is used to separate two chambers to observe trans-endothelial migration. Endothelial monolayers, however, are not able to recapitulate various functionalities, including full endothelial barrier properties, thus oversimplifying the 3D tumor microvasculature [18]. Alternatively, spheroids composed of endothelial and tumor cells can be seeded in 3D hydrogels, enabling endothelial cells to sprout and form hollow vascular structures within the spheroid. Tumor cells invading the vascular lumina could be visualized in this model [19]. Similarly, tumor cells seeded on top of a hydrogel containing lymphatic or vascular networks were utilized to visualize various stages of metastasis [20]. These models, however, do not allow the verification of the functionality of the formed endothelial vascular structures.

Recently developed cancer-on-a-chip models are evolving as powerful tools to investigate the tumor microenvironment [21] and its contribution to cancer intravasation and metastasis [[22], [23], [24], [25], [26], [27], [28], [29]]. They provide a controlled environment to simulate blood vessel-like conditions and enable the utilization of live-cell imaging to visualize intravasation events into artificial microvessels [30]. For instance, a dynamic microfluidic-based tumor-vessel co-culture system containing a vascular cavity with fluid flow was established to investigate the different phases of cancer metastasis and evaluate the effects of anti-cancer drug treatments [29]. In another microfluidic model, the adjacent culture of cancer associated fibroblasts (CAFs) in collagen type I hydrogels and endothelial monolayers (vessel analogs) demonstrated that CAF-secreted C-X-C motif chemokine ligand 12 (CXCL12) enhanced endothelial permeability [28]. Ni et al. developed a metastasis system composed of tumor cells separated from a microfluidic channel with fluid flow by a hollow lumen formed by a continuous endothelial monolayer [31].

Although current *in vitro* models have allowed the study of how various chemical and physical aspects influence the metastatic cascade, these models, similar to Transwell assays, consist of microchannels lined with a monolayer of endothelial cells. This does not fully recapitulate the blood vessel anatomy and lacks crucial features of functional microvessels, such as good physiologically representative vascular barrier functions [[22], [27], [32], [33]]. Other cancer on-a-chip models employed cancer spheroids embedded in a hydrogel holding microvascular networks (MVNs) [[34], [35], [36]]. These MVNs formed spontenously by vasculogenesis and as such exhibit improved barrier-properties, *in vivo*-like cell morphology and thus more physiologically representative functions [[37], [38]]. Nonetheless, most of the studies did not report any intravasation events, whereas one [34] relied on the natural shedding of cancer cells from spheroids into circulation. However, the time (7–12 days) and effort required to maintain the co-culture was substantial, and the latter model did not consider the process of EMT [34], thus rendering the study less physiologically relevant. In another similar microfluidic model, the center channel contained a spontenously formed large vessel-like structure [39], instead of MVNs. There, breast cancer cell intravasation could be visualized and quantified, demonstrating that the application of tumor necrosis factor-α (TNF- α) increased the percentage of intravasating tumor cells. Although the endothelial cells within the vessel-like structure exhibited proper cell-cell junctions, the vascular permeability coefficient was still one order of magnitude higher [39] than what was reported for microvasculature *in vivo* [40]. Hence, there is currently no physiologically relevant model that recapitulates both events of local invasion from the primary tumor via EMT, followed by intravasation into microvasculature.

Moreover, advanced machine learning models such as Cellpose [41], TrackFormer [42], and UNets [[43], [44], [45]] can provide pixel-perfect cell or tissue segmentation [45]. While advanced segmentation approaches have been used to quantify metastasis potential [[46], [47]], these studies have focused on readouts that include cell migration and spheroid protrusion dynamics, and do not directly assess intravasation events.

We have recently established a human microvasculature-on-a-chip model featuring stable, perfusable microvascular networks (MVNs) in microfluidic devices with physiologically relevant vascular barrier functions [48]. This is a prerequisite to faithfully model metastatic events, as cancer cells are required to breach the vascular barrier [49]. Utilizing this microvasculature-on-a-chip model, the objective of this research was thus to establish a human microphysiological *in vitro* model of EMT-driven lung cancer intravasation in a microfluidic device coupled with machine learning (ML)-assisted quantification of intravasation events within. To achieve this, we established a novel EMT induction cocktail (EMT-IC) based on the synergistic effects of macrophage conditioned medium and TGF-β1, inducing a robust migratory and invasive behaviour in lung cancer cells, outperforming standard EMT induction methods. This EMT-IC was then applied to a co-culture system of human lung cancer spheroids (microtumor masses) embedded within the human microvasculature-on-a-chip model in microfluidic devices [48]. Live cell imaging, high-resolution microscopy, and ML-assisted vessel segmentation combined with co-localization analysis to detect intravasation events were utilized to visualize and quantify intravasation events, respectively. The approach to combine MVNs with cancer spheroids in established 3-channel microfluidic devices is straightforward, thereby ensuring ease of use across different laboratory settings with high verifiability and reproducibility. When supplemented into microfluidic devices, the EMT-IC induced intravasation of lung cancer cells into the surrounding microvasculature, while the degree of which was dependent on the metastatic predisposition of the cancer cell line chosen. This emphasizes the universal applicability and sensitivity of our model, which is able to robustly assess cell-specific intravasation potential under quiescent and EMT-inducing conditions. Furthermore, this is the first study to visualize cancer intravasation into functional MVNs *in vitro* specifically driven by EMT and coupled with ML-assisted quantification. The accelerated intravasation timeline of 24 h, during which the established system maintains excellent reproducibility, represents a significant advantage, particularly for high-throughput applications. The model presented here opens avenues to study physiologically relevant lung cancer-related pathological processes in high spatiotemporal resolution and to utilize the established platform technology for drug development and high throughput screening.

## 2 Materials and methods

### 2.1 General cell culture

Human monocytic cell line THP-1 (ATCC TIB-202, Manassas, VA, USA) and NCI-H1975 cells (ATCC, Cat#. CRL-5908) were maintained in Roswell Park Memorial Institute (RPMI) 1640 Medium (GIBCO, Life Technologies, Grand Island, NY, USA, Cat#. 11875093), while A549 cells (ATCC, Cat#. CCL-185) and BEAS-2B cells (ATCC, Cat#. CRL-3588) were cultured in Dulbecco's Modified Eagle's Medium (DMEM) with 1 g/L glucose and GlutaMAX™ (GIBCO, Cat#. 10567014). Both culture media were supplemented with 1 % of 100 U/ml penicillin and 100 μg/ml streptomycin (P/S) (GIBCO, Cat#. 15140122), and 10 % fetal bovine serum (FBS) (ExCell Bio, Cat#. FSP500). Primary human umbilical vein endothelial cells (HUVECs, pooled) (ATCC, Cat#. PCS-100-013) and GFP-expressing HUVECs (TTFLUOR HUVECs) (Innport, Primera Planta, Spain Cat#. P20201) were cultured in Endothelial Growth Medium (EGM-2) (Lonza, Walkersville, MD, USA, Cat#. CC3162) up to passage 8. All cells were cultured at 37 °C in 5 % CO_2_ to 80 % confluency before passaging. HUVECs, A549, BEAS-2B and NCI-H1975 cells were cultured in tissue culture polystyrene flasks coated with 0.1 % gelatin (Sigma-Aldrich, Saint Louis, MO, USA, Cat#. G1890). HUVECs, A549, BEAS-2B, NCI-H1975 and phorbol 12-myristate 13-acetate (PMA)-treated THP-1 induced macrophages were trypsinized using TrypLE™ Express (GIBCO, Life Technologies,Cat#. 12605010) for 3 min at 37 °C and resuspended in EGM-2, DMEM with 10 % FBS and 1 % P/S and RPMI with 10 % FBS and 1 % P/S, respectively.

### 2.2 Generation of macrophage-conditioned medium (M**ϕ**-CM)

One million THP-1 cells were first seeded per well of a 6-well plate containing 3 ml of RPMI-1640 medium with 10 % FBS, 1 % P/S, and 100 ng/ml of PMA (STEMCELL Technologies, Vancouver, Canada, Cat#. 74042) to induce macrophage differentiation. After 24 h, the attached cells were washed with phosphate-buffered saline (PBS) before changing to 3 ml of EGM-2 medium and incubated for 48 h. Subsequently, the supernatant was extracted and filtered through a 0.22 μm filter before being diluted 1:1 with fresh EGM-2 medium. The M**ϕ**-CM was stored at −80 °C and thawed right before use.

### 2.3 Proteome profiler cytokine array

M**ϕ**-CM was analyzed for its cytokine composition using the Proteome Profiler Human XL Cytokine Array Kit (R&D Systems, Minneapolis, USA, Cat#. ARY022B). 105 cytokines can be simultaneously detected based on the manufacturer's instructions using antibodies and reagents from the kit. Signals in the cytokine array membrane were captured using the ChemiDoc™ MP Imaging System (Bio-Rad Laboratories, CA, USA, Cat#. 17001402). To quantify the difference in the level of cytokines between control (unconditioned EGM-2 culture medium) and M**ϕ**-CM, the average pixel intensity of each duplicate spot was measured using ImageJ v1.54f software [50]. Cytokines were classified either as EMT inducers or EMT suppressors, and either as pro-inflammatory or anti-inflammatory cytokines, then plotted as a log_2_ fold change of M**ϕ**-CM to control using GraphPad Prism 9.4.1 software.

### 2.4 Spheroid formation

500-cell A549, NCI-H1975 or BEAS-2B cell spheroids were formed using Aggrewell™ 400 plates (STEMCELL Technologies, Cat#. 34411) according to the manufacturer's instructions. Briefly, 500 μL of anti-adherence rinsing solution (STEMCELL Technologies, Cat#. 07010) was added into each well and centrifuged at 1300×*g* for 5 min to prevent cell adhesion. After removing the rinsing solution, 600,000 cells of lung cancer cells suspended in 2 ml of DMEM with 10 % FBS and 1 % P/S were added into each well to achieve the targeted 500-cell spheroids in each microwell. Cells were aggregated by centrifugation at 100×*g* for 3 min and were incubated for 3 days before being flushed out for subsequent experiments.

### 2.5 Cell seeding for 2D culture

Post-trypsinized A549, NCI-H1975 or BEAS-2B cells were seeded in tissue culture-treated 48-well plates at 15,000 cells/cm^2^ and left overnight for attachment. After overnight incubation, the culture medium was replaced with either control (EGM-2 medium) or the following experimental media: (i) EGM-2 medium with 5 ng/ml of recombinant human TGF-β1 (PeproTech, New Jersey, USA, Cat#. 100-21); (ii) EGM-2 with M**ϕ**-CM; and (iii) EGM-2 with M**ϕ**-CM and TGF-β1. This is followed by incubation for 2 days at 37 °C before further analysis.

### 2.6 Analysis of A549 cell morphology

Phase contrast images of A549 were taken with a 10× objective with a Nikon ECLIPSE Ti2-A inversion fluorescence microscope (Nikon, Tokyo, Japan). Circularity of cells was assessed via ImageJ software. The circularity of 1.0 indicated a perfect circle while values approaching 0.0 represented a less circular or more elongated cell morphology.

### 2.7 Viability assay

A549 cells subjected to different conditions were investigated for cellular viability by two approaches. First, the number of cells per field of view was counted based on nuclear staining by 4′,6-diamidino-2-phenylindole (DAPI) (Thermofisher, Massachusetts, USA, Cat#. 62247). In addition, Live/Dead Assay was performed using Live/Dead viability/cytotoxicity kit (Invitrogen, Waltham, Massachusetts, USA, Cat#. L3224) according to the manufacturer's instructions. Dead and live cells were stained with 4 μM ethidium homodimer-1 and 2 μM calcein acetoxymethyl ester, respectively, for 15 min at 37 °C, prior to imaging with a Nikon ECLIPSE Ti2-A inversion fluorescence microscope with 10× objective.

### 2.8 Immunocytochemistry for 2D culture

A summary of the sources of antibodies and reagents used in this study is presented in Table 1. Cell fixation was first performed using 4 % paraformaldehyde (PFA) (Thermo Scientific, Cat#. 5735) followed by permeabilization with 0.1 % Triton™ X-100 (Sigma-Aldrich, Saint Louis, USA, Cat#. T9284) for 15 min and 1-h blocking with 3 % bovine serum albumin (BSA) (Sigma-Aldrich, Saint Louis, USA, Cat#. A7906) in PBS. The cells were then incubated overnight with primary antibodies in PBS with 1 % BSA. Upon overnight incubation, cells were washed thrice with PBS and incubated with secondary antibodies in PBS with 1 % BSA for 1 h, followed by counterstaining with DAPI in PBS for 10 min. Fluorescent stained E-cadherin and vimentin were visualized using Nikon ECLIPSE Ti2-A with 10× objective and the fluorescence intensity was calculated using ImageJ v1.54f software with the following formula [51]:

**Table 1:** List of antibodies and reagents for immunocytochemistry in 2D.

Reagents	Dilution	Supplier	Cat#.
Mouse monoclonal anti-E-cadherin	1:500	Thermo Fisher	13–1700
Mouse monoclonal anti-Vimentin	1:500	Thermo Fisher	MA5-11883
Goat anti-mouse-IgG AF 555	1:500	Abcam	ab150118
4′,6-diamidino-2-phenylindole (DAPI)	1:1000	Thermo Fisher	62247

Corrected total cell fluorescence (CTCF) = integrated density – (area of selected cell × mean background fluorescence intensity)

### 2.9 Gene expression analysis

RNA extraction and cDNA synthesis were performed using RNAiso Plus (Takara Bio Inc., Cat. #9109) and PrimeScript™ RT Master Mix (Takara Bio Inc., Cat. #RR036A), respectively, following the manufacturer's protocols. For RT-qPCR analysis, 50 ng of cDNA and target-specific primers (Table 2) were amplified with ChamQ SYBR Color qPCR Master Mix (Vazyme, Cat. #Q411-03) on a QuantStudio™ 7 Pro Real-Time PCR System (Applied Biosystems™, Carlsbad, CA, USA). Gene expression levels were quantified using the cycle threshold (ΔΔCT) values which were normalized to housekeeping gene GAPDH and the relative fold change was compared to A549 control condition (EGM2 only) collected 24 h after medium change. Another alternative housekeeping gene *B2M* was used to ensure the validity of *GAPDH*, which yielded similar results.

**Table 2:** Primer sequences used in RT-qPCR.

Gene	Forward Primer	Reverse Primer
ZEB1	5′-GGCATACACCTACTCAACTACGG-3′	5′-TGGGCGGTGTAGAATCAGAGTC-3′
ZEB2	5′-AATGCACAGAGTGTGGCAAGGC-3′	5′-CTGCTGATGTGCGAACTGTAGG-3′
SNAI1	5′-TGCCCTCAAGATGCACATCCGA-3′	5′-GGGACAGGAGAAGGGCTTCTC-3′
SNAI2	5′-ATCTGCGGCAAGGCGTTTTCCA-3′	5′-GAGCCCTCAGATTTGACCTGTC-3′
TWIST1	5′-GCCAGGTACATCGACTTCCTCT-3′	5′-TCCATCCTCCAGACCGAGAAGG-3′
B2M	5′- CCGTGTGAACCATGTGACTT-3′	5′- CCAATCCAAATGCGGCATCT-3′
GAPDH	5′- CCAGGGCTGCTTTTAACTCTGGTAAAGTGG-3′	5′-ATTTCCATTGATGACAAGCTTCCCGTTCTC-3′

### 2.10 Cell migration assay

A549 cells were cultured in 48-well plate wells until confluency. The culture medium in each well was first replaced with PBS, upon which the confluent layer of A549 cells was scratched using a P1000 pipette tip to create a gap. Floating cells were removed by washing with PBS, before the initial culture medium was added back. Phase contrast imaging was subsequently performed at 0-, 6-, and 12-h time points using Nikon ECLIPSE Ti2-A inversion fluorescence microscope with 10× objective. The gap area at specific time points was quantified using ImageJ v1.54f software.

### 2.11 Spheroid invasion assay

A549 spheroids were centrifuged and resuspended in different conditions at a density of 200 spheroids/ml. For each well of 48 well plate, 75 μL of spheroid suspension, which was estimated to contain 15 spheroids, was mixed 1:1 with 75 μL of 2 mg/ml neutralized collagen type I TeloCol®-6 hydrogel (Advanced BioMatrix, Carlsbad, USA, Cat#. 5225). Collagen type I hydrogels embedded with spheroids were incubated for 2 h at 37 °C to achieve polymerization before being overlaid with 300 μl of medium. EGM-2 medium was used as control. The spheroids were incubated for 24 h to investigate their invasion ability in different conditions. After 4 % PFA fixation for 30 min, the spheroids were imaged using Nikon ECLIPSE Ti2-A inversion fluorescence microscope with 10× objective. To quantify the invasion ability of the spheroids, the area of invasion was manually traced and measured using ImageJ v1.54f software and divided by the initial spheroid area.

### 2.12 Device fabrication

Microfluidic devices were fabricated by replica molding on a silicon wafer and soft lithography using PDMS (polydimethylsiloxane) [[48], [52]]. Briefly, a 100 μm layer of SU-8 3050 negative photoresist (Kayaku Advanced Materials, Massachusetts, USA) primer was spin-coated on a silicon wafer before being exposed to a photomask exhibiting the negative pattern of the channel structures designed by computer aided designs (CAD) for photolithography. The SU8 was then exposed to UV light (set as 20 mW/cm^2^ at 365 nm) for 45 s, followed by pattern developing. PDMS and curing agent (Sylgard 184, Dow Corning, Michigan, USA) were mixed at 10:1 (w/w) ratio and cast onto the SU8 master. After thermal curation at 60 °C for 2 h, a positive replica-molded pattern on PDMS was separated from the wafer. Patterned PDMS was cut into individual devices and inlet and outlet ports were punched using 1- and 3-mm biopsy punchers. Next, the devices and glass slides were cleaned with 100 % ethanol, water, and dried with a nitrogen gas air gun before being treated with oxygen plasma (Harrick Plasma, New York, USA) for 45 s to create covalent bonding between the glass slide and the PDMS device. Immediately after the PDMS pieces and glass slides were assembled, the channels were coated with 1 mg/ml of poly-L-lysine (PLL) (Molecular weight: 30,000–70,000) (Meryer, Shanghai, China, Cat#. 25988-63-0) dissolved in water for at least 20 min before autoclaving. PLL coating increased the hydrophilicity of the device enabling easy loading of the hydrogel.

### 2.13 Device design

A previously established microfluidic design with a 3-channel system was utilized [[48], [52]]. The channels have a height of 100 μm and length of 14.5 mm with triangular posts separating the center hydrogel channel from the adjacent media channels. The posts are 100 μm apart. The width of the hydrogel channel and media channels is 1300 μm and 500 μm, respectively. Media inlet ports and cell inlet ports have a diameter of 1000 μm and 500 μm, respectively (Fig. 3A).

### 2.14 PDMS device seeding

A solution of fibrinogen (Sigma-Aldrich, Cat#. F8630-5G) was prepared by dissolution in PBS at 15 mg/ml freshly for each experiment. Thrombin solution (Sigma-Aldrich, Cat#. T4648) was prepared in 1 % (w/v) BSA (Sigma-Aldrich, Cat#. A7906) in PBS solution at 100 U/ml and stored in aliquots at −20 °C. HUVECs were resuspended in EGM-2 containing 6 U/ml of thrombin, the cell solution was mixed with fibrinogen solution at 1:1 ratio to achieve final concentrations at 7.5 mg/ml for fibrinogen, 3 U/ml for thrombin and 6 × 10^6^ cells/ml HUVECs. 300-cell A549 spheroids were formed as described under “spheroid formation” previously. The spheroids were collected and resuspended in 200 μl of EGM-2 and 2 μl/droplets were pipetted onto a Petri dish for visualization and selection. Droplets with more than 10 spheroids were selected to be introduced quickly into the center channel with the mixture of HUVECs, fibrinogen solution as well as thrombin, and the device was placed at 37 °C in a humidified incubator for 1 h to allow the fibrinogen to be polymerized by thrombin into a fibrin hydrogel. Next, EGM-2 was added into the medium channels. Medium was changed daily. To introduce macromomolecular crowding (MMC) into 3D cell culture, media containing Ficoll macromolecules (25 mg/ml Ficoll 400 - Cytiva, Marlborough, MA, USA, Cat#. 17-0300-50; and 37.5 mg/ml of Ficoll 70 - Cytiva, Cat#. 17-0210-10), were introduced from day 1 onwards. The composition of the Ficoll cocktail was optimized as previously described [39], exhibiting a calculated fractional volume occupancy (FVO) of 17 % and was demonstrated to enhance ECM deposition and basement formation around MVNs. On day 3, the co-culture was incubated with or without EMT-inducing cocktail also supplemented with 25 mg/ml of Ficoll 400 and 37.5 mg/ml of Ficoll 70 for 24 h.

### 2.15 Microvascular network permeability assay and quantification of permeability coefficient

The permeability of MVNs was assessed by introducing fluorescein-5-isothiocyanate polyvinylpyrrolidone (FITC-PVP) (250 μg/ml in EGM-2 solution; Invitrogen, Cat# A23017) into one of the medium channels. Prior to this, an endothelial monolayer was established in one medium channel on day 2 of culture. On day 4, the medium was removed from each port and replaced with 15 μl of FITC-PVP solution added specifically to the outlet port of the endothelial cell-seeded channel. Perfusion of the MVNs was achieved through hydrostatic pressure generated by maintaining different medium levels in the two adjacent channels until equilibrium was established.

Fluorescence imaging was conducted using a Nikon Ti2-E inverted fluoresecence microscope, with time-lapse images captured every 15 s over a 15-min period. The permeability coefficient was calculated according to established methods [53], based on several key assumptions: the fluorescence intensity within vessels remained constant, the vessels maintained cylindrical morphology, and the rate of fluorophore efflux from vessels equaled its accumulation rate in the surrounding hydrogel.

For accurate permeability measurements, analysis windows were carefully positioned to include both the vessel lumen and adjacent hydrogel regions. Only vessels with diameters smaller than 50 μm were selected to ensure circular cross-sections, a critical requirement for the permeability calculation formula. Additionally, measurement locations were chosen to avoid any artifacts from direct dye diffusion from the side channels into the hydrogel. The permeability coefficient was ultimately derived from the changes in average fluorescence intensity between initial and final time points using the prescribed equation.

*I*_*i*_, *I*_*b*_ and *I*_*f*_ correspond to the mean fluorescence intensities within the measurement window at the initial time point, background level, and final time point, respectively. The time interval between measurements is denoted as Δt, while d represents the mean vessel diameter within the measurement window.

$$P\left(\frac{cm}{s}\right)=\frac{1}{I_{i}-I_{b}}\left(\frac{I_{f}-I_{i}}{\Delta t}\right)\times \frac{d}{4}$$

### 2.16 Immunocytochemistry for 3D culture

After 4 days of culture, the devices were washed with PBS and the cultures fixed with 4 % PFA through the medium channels for 15 min, then permeabilized with 0.25 % Triton X-100 in PBS for 10 min. After blocking with 5 % BSA in PBS for 1 h, samples were incubated with the respective primary antibodies (see Table 3) in PBS containing 0.5 % BSA for 16 h at 4 °C. Samples were then washed three times with PBS for 5 min each, before being incubated with secondary antibodies (see Table 3) in PBS containing 0.5 % BSA for at least 3 h at room temperature. The samples were washed and stored in PBS at 4 °C before imaging.

**Table 3:** List of antibodies and reagents for immunocytochemistry in 3D.

Reagents	Dilution	Supplier	Cat#.
Mouse monoclonal anti-VE-cadherin	1:200	Santa Cruz	Sc-9989
Mouse monoclonal anti-Laminin	1:200	Abcam	ab77175
Mouse monoclonal anti-CD31	1:200	Abcam	ab9498
Rabbit monoclonal anti-Vinculin	1:200	Abcam	ab155120
Anti-mouse-AF-555	1:200	Abcam	ab150118
Anti-mouse-AF-488	1:200	Abcam	ab150113
Anti-rabbit-AF-647	1:200	Abcam	ab150075
Phalloidin-iFluor 647	1:700	Abcam	ab176759
Phalloidin-iFluor 555	1:700	Abcam	ab176756
4′,6-diamidino-2-phenylindole (DAPI)	1:700	Thermo Fisher	62247

### 2.17 Microscopy

Confocal imaging was performed using an inverted confocal microscope (Leica SP8, Leica Microsystems, Germany) with 20x and 63× objectives. Images of laminin, VE-cadherin and vinculin were taken and visualized. Orthogonal projections of the z-stack images were reconstructed to visualize events of cancer intravasation. All images were analyzed with ImageJ v1.54f software (https://imagej.nih.gov/ij/). Additionally, visualization of the MVNs in proximity of cancer spheroids was performed using Qiber3D [54].

### 2.18 Quantification of MVN vascular junction and tubule length per field of view (FOV)

Phase contrast images (10x) of MVNs were analyzed using ImageJ v1.54f software and Angiogenesis Analyzer plugin 1.0 [55] (https://imagej.nih.gov/ij/macros/toolsets/Angiogenesis%20Analyzer.txt). Briefly, raw images were converted into binary images using automated thresholds for binary tree analysis in the Angiogenesis Analyzer plugin. The number of junctions and total branching lengths were measured and presented as vascular junction number and tubule length per FOV, respectively. Junctions were denoted by points with at least 3 neighbors, and tubule length referred to the length of elements bound by two junctions or between one junction and one endpoint. The Angiogenesis Analyzer plugin has been widely adopted by researchers for quantitative assessment of vascular network parameters, including node count, master junctions, total vessel length, and tube formation efficiency in angiogenesis assays [[56], [57], [58]]. This plugin also reduces bias during quantification.

### 2.19 Western blotting

PDMS devices were peeled off from the glass slide using a cutter and cells within the center channel were lysed using a 1:1 mixture of 2x Laemmli buffer and 2x protease inhibitor cocktail (Sigma-Aldrich, Cat#. P8340). Protein concentration of collected samples were measured using Pierce™ Rapid Gold BCA Protein Assay Kit (Thermo Fisher, Cat#. A53226). Samples were denatured at 95 °C for 5 min and loaded at equal protein amounts into 8 % SDS-polyacrylamide gels (Life Technologies, Cat#. HC2040) and subjected to electrophoresis at 120 V. After protein separation, samples were electrotransferred to a polyvinylidene difluoride (PVDF) membranes (Thermo Scientific, Cat#. 88518) using a Power Blotter XL (Life Technologies, Cat#. PB0013). For membrane staining, membranes were incubated with 5 % skimmed milk (Phygene Biotechnology Co Ltd, FuZhou, China, Cat#. PH1519) in TBS-Tween 20 (TBST), containing 50 mM Tris, 150 mM NaCl and 0.05 % Tween 20 (Sigma-Aldrich, Cat#. P2287) to block non-specific antibody binding before incubation with primary antibodies (see Table 4) in TBST containing 1 % skimmed milk at 4 °C overnight. After washing three times with TBST, secondary antibodies (see Table 4) resuspended in TBST containing 1 % skimmed milk were added to the blots for 1 h at room temperature. After washing three times with TBST, protein bands were then detected with ECL Super Signal West Pico Plus (Life Technologies, Cat#. 34580) using ChemiDoc™ MP Imaging System and quantified by Image Lab 6.1 software (Bio-Rad).

**Table 4:** List of antibodies and reagents for western blotting.

Reagents	Dilution	Supplier	Cat#.
Mouse monoclonal anti-VE-cadherin	1:500	Santa Cruz	Sc-9989
Mouse monoclonal anti-Laminin	1:500	Abcam	ab77175
Anti-mouse-HRP	1:5000	Abcam	ab205719
Rabbit Anti-GAPDH	1:5000	Abcam	ab181602
Anti-Rabbit-HRP	1:5000	Abcam	ab6721

### 2.20 Live cell imaging

A549 and HUVECs were resuspended and pre-labelled with PKH26 Red Fluorescent and PKH67 Green Fluorescent Cell Linker Midi Kit for General Cell Membrane Labeling according to the manufacturer's protocol (Sigma-Aldrich, Cat#. MIDI26, MIDI67). The 24-h live cell imaging was started on day 3 of culture and after the optional addition of an EMT-inducing cocktail to trace any events of cancer intravasation using a Leica Mica confocal microscope (Leica Mica, Leica Microsystems, Germany) with 20× objective. To quantify the rate of intravasation from the fluorescent images, the number of cancer cells localizing with the green fluorescence of MVNs was manually counted.

### 2.21 Machine learning-assisted vessel segmentation and quantification of intravasation events

We defined the localization of fluorescently labelled A549 cancer cells within the MVN as a proxy for cancer cell intravasation. All analyses were performed at the 24-h time point of the fluorescent live cell images using Fiji (ImageJ v1.54f) [50].

Vessel Segmentation: Vessel segmentation was achieved through a machine learning-assisted approach on stacks of phase contrast and GFP channel images. This dual-channel combination enabled reliable segmentation even when GFP expression in HUVECs decreased during culture. Initially, a default threshold was applied to the GFP channel to segment the HUVECs. The Trainable Weka Segmentation (TWS) plugin [59] was then trained using 28 images. Segmentation parameter settings include trainable features like Gaussian blur, Hessian, membrane projections, Sobel filter, and difference of Gaussians. The parameters are set as follows: membrane thickness of 1, patch size of 19, minimum sigma of 1.0, and maximum sigma of 16.0. The chosen classifier option is “fast random forest” using 200 trees and a batch size of 100. These settings determine how TWS learns and applies machine learning for image segmentation. Binary semantic segmentation was performed on images containing vessels and spheroids, with vessels classified as one class while spheroids and background were classified as another class.

Tumor Spheroid Extraction: Each microscopy image used for quantification contained multiple tumor spheroids present on one microfluidic chip. A square ROI was drawn around each tumor region (tROI) to extract each spheroid separately. The Analyze Particles plugin was utilized to locate each spheroid, and the size of the tROI was set to 115 % of the major axis after fitting an ellipse to the spheroid, ensuring only the signal in its immediate proximity was included for analysis.

Segmentation Workflow: MVNs were segmented by applying a default threshold to the GFP channel and subsequently applying the Weka classifier to the phase contrast and binarized GFP channels for each tROI (Fig. 6C). Morphological operations, such as Erosion and Dilation, were utilized to reduce imaging artifacts. The Analyze Particles plugin was then used to generate an ROI of the segmented vessels (vROI). This vROI was overlaid onto the channel containing the pre-fluorescently labelled A549 cancer cells. Fluorescence signals outside the vROI were excluded (Fig. 6C), and an Otsu threshold [60] was applied to segment the cancer particles inside the vROI. The fluorescent particles of A549 cells inside the segmented vessels were counted (minimum size: 50 μm^2^, circularity 0.5–1.0).

ImageJ scripts were developed to facilitate high-throughput automated image analysis and are available at: https://github.com/anna-jaey/FijiScriptToolbox?tab=readme-ov-file#particle-counting-and-area-ratio-quantification-in-multi-channel-fluorescence-images.

### 2.22 Data presentation and statistical analysis

At least three independent biological runs with at least three replicates each were performed for each experiment. Levene's test was conducted to test for homogeneity of variances across samples. For parametric samples, unpaired *t*-test and one-way ANOVA followed by post hoc Tukey test was performed for samples with two and three or more conditions, respectively. On the other hand, non-parametric samples with two and three or more conditions were tested with Mann-Whitney test and Kruskal-Wallis test, respectively. Data were shown as means ± standard deviation each containing at least three replicates, and statistical significance was set as follows: ∗p < 0.05; ∗∗p < 0.01; ∗∗∗p < 0.001; ∗∗∗∗p < 0.0001. All micrographs were quantified using ImageJ v1.53t software (https://imagej.nih.gov/ij/) and all analyses were performed using GraphPad Prism v9.3.1 (GraphPad Software, San Diego, CA, USA, www.graphpad.com). All schematics were created with bioRender (www.biorender.com).

## 3 Results

### 3.1 M**ϕ**-CM and TGF-β1 synergistically facilitate EMT in A549 cells

EMT is characterized by loss of apical-basal polarity, disruption of cell-to-cell adhesion and increased migration and invasion abilities of cancer cells into surrounding tissues [61]. Standard EMT induction protocols utilize TGF-β1, as TGF-β1/SMAD serves as the EMT hallmark pathway resulting in the upregulation of EMT-related transcription factors [62]. Nonetheless, the induction of EMT using a single growth factor often results in an incomplete transition and other EMT drivers have been considered. Physiologically, cellular components in the tumor microenvironment, such as CAFs and tumor-associated macrophages (TAMs), have been implicated to have decisive roles in the EMT of cancer cells [63]. Indeed, the secretome of TAMs was shown to contain a variety of cytokines that are involved in EMT induction [[63], [64], [65], [66], [67]], and macrophages or their conditioned medium have been demonstrated to promote EMT in epithelial cancer cells [[68], [69], [70], [71]]. Both, pro-inflammatory (M1) and immunomodulatory (M2) polarized macrophages have been implicated in driving EMT [72].

**Fig. 1:**
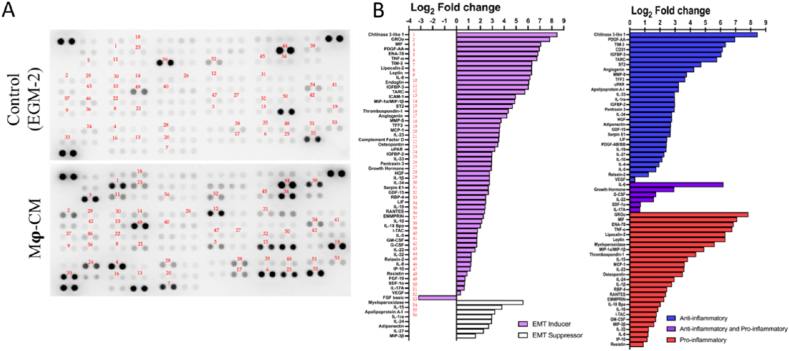
**Cytokine profiling of Mϕ-CM by a proteome profiler array revealed an abundance of EMT-inducers secreted by THP-1-derived Mϕ.** (A) Images of protein arrays of EGM-2 (unconditioned culture medium) and M**ϕ**-CM with chemiluminescently visualized signals for various secreted components. (B) The levels of selected cytokines in M**ϕ**-CM were quantified, and levels were plotted as Log_2_ fold-changes compared to control and classified according to their role either as EMT inducers or EMT suppressors, and either as pro- and/or anti-inflammatory cytokines.

Adenocarcinomic human alveolar basal epithelial cells (A549) were exposed to TGF-β1 and macrophage-conditioned medium (M**ϕ**-CM) or the combination of both and investigated for their ability to undergo EMT. M**ϕ**-CM was derived from THP-1-derived macrophages. The composition of the M**ϕ**-CM was analyzed using a proteome profiler array. Compared to the unconditioned EGM-2 culture medium, M**ϕ**-CM was enriched in a wide array of known EMT-inducers, which outweighed the number of EMT-suppressors. At the same time the number of factors identified as pro-inflammatory and/or anti-inflammatory was relatively balanced (Fig. 1), suggesting M**ϕ**-CM to contain a suitable cocktail for EMT induction in cancer cells.

**Fig. 2:**
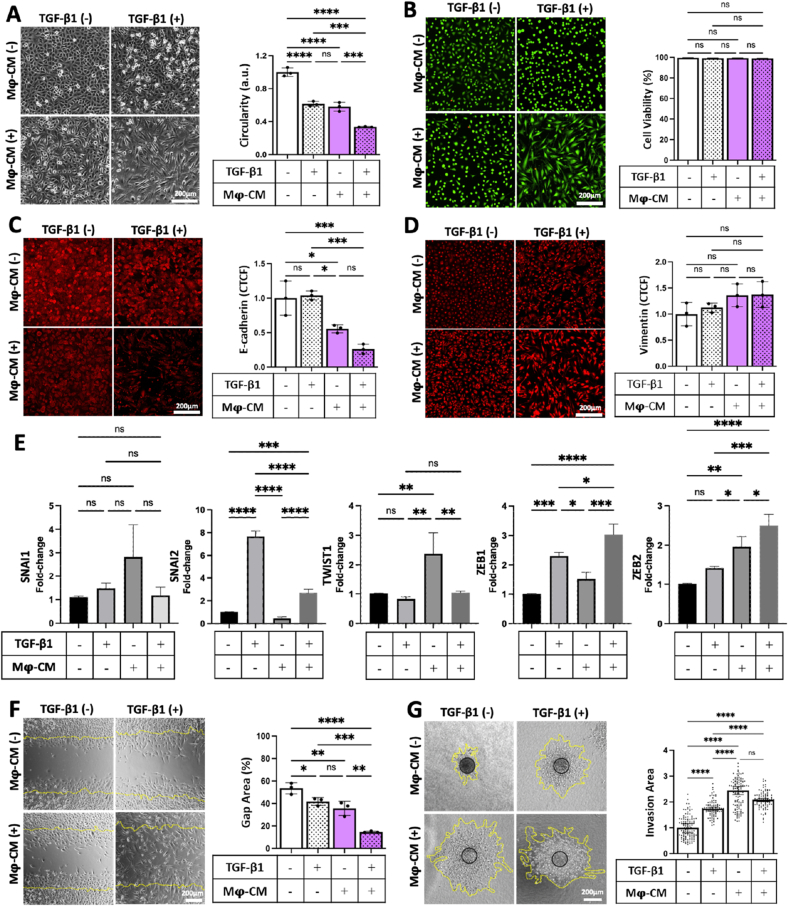
**Mϕ-CM and TGF-β1 synergistically drove EMT in A549 cells.** A549 cells were incubated with TGF-β1, M**ϕ**-CM or combination of both for two days. (A) Representative micrographs depict cellular morphology. Images were analyzed for cellular circularity by ImageJ software, which was plotted after being normalized to the untreated control. (B) Images of Live/Dead staining of treated A549 and quantification of cellular viability after 2 days of incubation with various EMT-inducers. (C) Representative micrographs of A549 cells immunostained for epithelial marker E-cadherin and (D) mesenchymal marker vimentin. Fluorescence intensity of signals for E-cadherin and vimentin was determined by measuring the Corrected Total Cell Fluorescence (CTCF), which was then normalized to respective controls. (E) Relative gene expression of SNAI1, SNAI2, TWIST1, ZEB1 and ZEB2 was determined by real-time qPCR. The relative mRNA levels of each gene (ΔΔCT values) were normalized to the respective levels of GAPDH (housekeeping gene). (F) Representative micrographs of a 2D scratch migration assay. Dotted yellow lines indicate the initial gap area. Percentage of initial gap area free of cells after 12 h of treatment was quantified by ImageJ software. (G) 3D invasion assay of A549 spheroids embedded in collagen type I hydrogels after 24 h of incubation. The degree of invasion of A549 cells into their surrounding environment was defined as the total area of invasion (invading cellular front is indicated by yellow dotted line) divided by initial area of the spheroid (indicated by black circle) and normalized to control.∗, p < 0.05; ∗∗, p < 0.01; ∗∗∗, p < 0.001; ∗∗∗∗, p < 0.0001. n = 3 biological replicates.

A549 cells were thus incubated with the standard EMT-inducing cytokine TGF-β1, M**ϕ**-CM, or a combination of both, to establish the best formulation of an EMT-IC. Morphologically, A549 cells exhibited a significantly decreased cellular circularity when exposed to TGF-β1 or M**ϕ**-CM, while the combination of both induced the most observable change from cubic to spindle shape morphology (Fig. 2A). While none of the treatments affected cellular viability (Fig. 2B), the number of cells per field of view decreased (Suppl. Fig. 1), which was attributed to the aforementioned changes of cell morphology to a larger spindle-like shape. Simultaneously, A549 cells exposed to TGF-β1 did not exhibit a visible decrease of epithelial marker E-cadherin, while A549 cells treated with M**ϕ**-CM or the combination of both experienced the most significant reduction of E-cadherin signal (Fig. 2C). An upregulation, albeit not significant, in mesenchymal marker vimentin was detected in A549 cells exposed to the combination of TGF-β1 or M**ϕ**-CM (Fig. 2D). Gene expression analysis revealed that an overall upregulation of key genes involved in EMT, when A549 cells were exposed to the combination of M**ϕ**-CM and TGF-β1 (Fig. 2E). Although SNAI1 gene expression exhibited a relatively high variability between treatment groups, SNAI2 was significantly upregulated in A549 treated with TGF-β1 alone or in combination with M**ϕ**-CM. TWIST1 was only significantly upregulated in A549 cells treated with M**ϕ**-CM, while ZEB1 and 2 exhibited highest expression in A549 cells treated with the combination of both (Fig. 2E). As marker expression and morphological changes are insufficient to confirm successful EMT, the functionality of the transformed A549 cells in terms of migration and invasion potential was investigated. Individually, TGF-β1 and M**ϕ**-CM enhanced the migratory potential of A549 to a similar extent, while the combination of both more than doubled this effect (Fig. 2F). Next, A549 spheroids (tumor micromasses formed in microwells (Suppl. Fig. 2) were embedded in 3D collagen type I hydrogels to measure the ability of tumor cells to invade into their local environment. Treatment with TGF-β1 increased their invasiveness, while treatment with M**ϕ**-CM or the combination of both resulted in comparable and most advanced invasion into the surrounding environment (Fig. 2G). Taken together, the combination of TGF-β1 and M**ϕ**-CM induced the most effective EMT in A549 cells and served as the EMT-IC in all subsequent experiments.

**Fig. 3:**
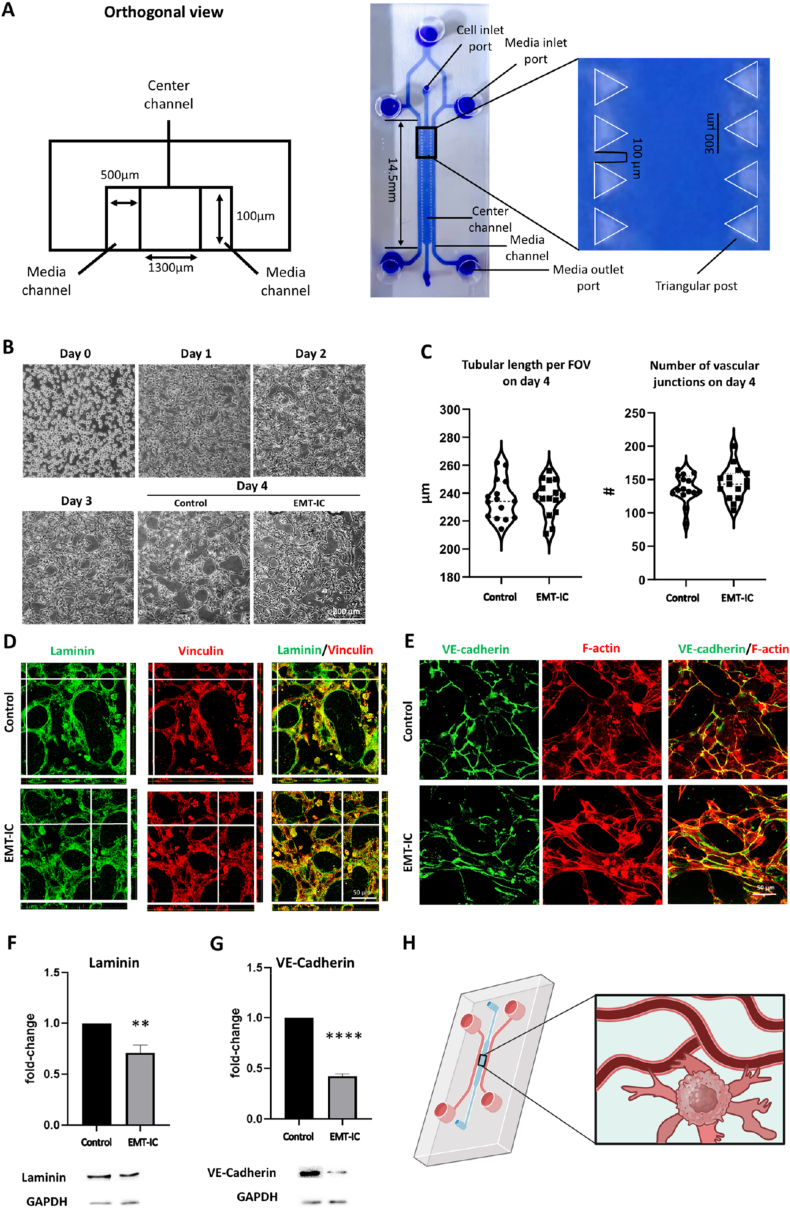
**The effect of EMT-IC (TGF-β1 & Mϕ-CM) on the stability and integrity of MVNs.** After 24 h of incubation with EMT-IC or control medium (unconditioned EGM-2 lacking TGF-β1) MVNs were immunostained for laminin, vinculin and VE-cadherin. F-actin was visualized by phalloidin staining. (A) Photograph and dimensions of the three-channel microfluidic device. (B) Representative micrographs showing the formation of MVNs by HUVECs in microfluidic devices with and without the introduction of EMT-IC on day 3 for 24 h. (C) Quantification of total tubular length per field of view (FOV) and number of vascular junctions using ImageJ angiogenesis analyzer plug-in. (D) Confocal microscopy Z-stack images of co-stained laminin and vinculin displayed in orthogonal view. Locations of cross-sections are indicated by white lines and orthogonal cross-sections are displayed on the sides. (E) Confocal microscopy Z-stack images of co-stained VE-cadherin and F-actin displayed as maximum projections. (F) Western blot and densitometric band analysis of laminin and (G) VE-cadherin, normalized to their respective GAPDH levels of samples collected on day 4. Protein levels are displayed as fold-change and compared to control. (H) Schematic diagram of the cancer-on-a-chip model. Generated by BioRender. ∗∗, p < 0.01; ∗∗∗∗, p < 0.0001. n = 3 biological replicates.

### 3.2 EMT-IC did not adversely affect the stability of MVNs in microfluidic devices

Using an established three channel microfluidic device (Fig. 3A), HUVECs were seeded in fibrin hydrogels into the center channel, where they formed stable MVNs by vasculogenesis [48]. On day 3, the culture medium was changed to EMT-IC or control medium (unconditioned EGM-2 lacking TGF-β1), and MVNs were incubated for an additional 24 h (Fig. 3B). MVNs formed consistently and reproducibly across various devices and biologic repeats, ensuring reproducibility and robustness of the model (Suppl. Fig. 3). The EMT-IC had no apparent effects on MVN stability, as shown in the quantification of the total length of tubular structures, as well as the number of vascular junctions (Fig. 3C). Co-staining of major basement membrane component laminin and focal adhesion marker vinculin demonstrated that endothelial structures were enveloped in a tight sheath of basement membrane, suggesting that the EMT-IC had no adverse effects on the apical-basal polarity of microvessels, with cellular focal adhesions closely interacting with vascular basement membrane (Fig. 3D). Co-staining of vessel-specific endothelial adhesion molecule VE-cadherin and F-actin enabled the visualization of cell-cell junctions respective to the overall structure of MVNs. Clearly outlined cell borders were visualized in both conditions, albeit appearing more tethered in MVNs incubated with EMT-IC (Fig. 3E). Semi-quantification of laminin levels, as determined by western blotting, suggested a decrease of basement membrane levels in MVNs exposed to EMT-IC (Fig. 3F), while western blotting for VE-cadherin revealed a 50 % decrease in protein levels in MVNs exposed to EMT-IC (Fig. 3G).

### 3.3 EMT-IC did not adversely affect the stability of MVNs when co-seeded with A549 spheroids in microfluidic devices

**Fig. 4:**
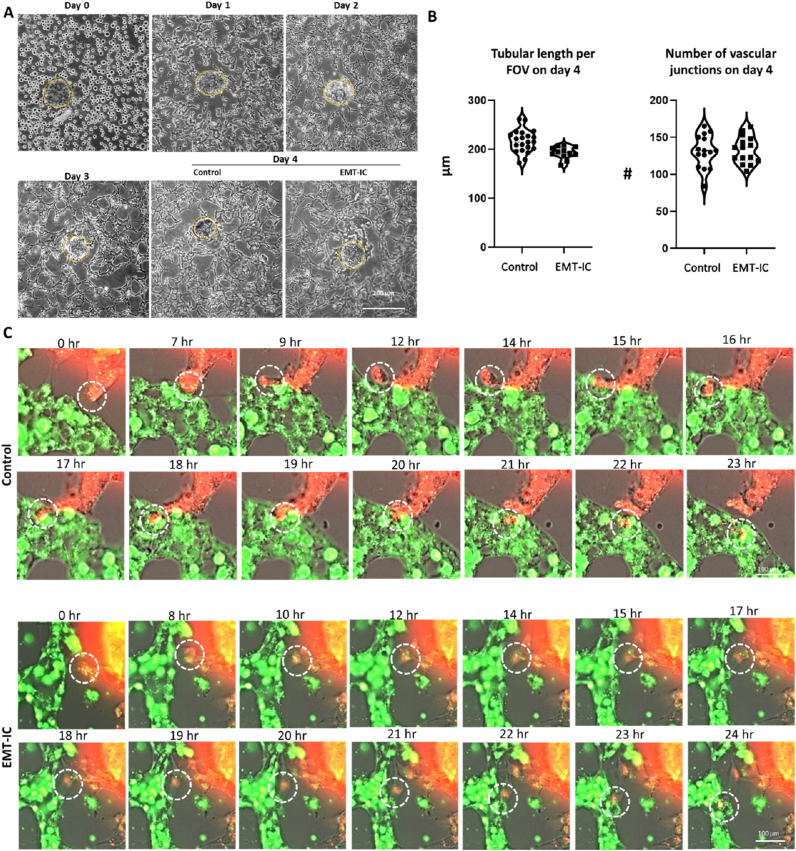
**Live cell fluorescent imaging of EMT-IC (TGF-β1 with Mϕ-CM)-facilitated A549 cancer cell migration and intravasation into MVNs over 24 h.** (A) Representative micrographs showing the formation of MVNs in the presence of A549 spheroids in microfluidic devices with and without the introduction of EMT-IC on day 3 for 24 h. Initial tumor masses are outlined by yellow dashed circles. (B) Quantification of total tubular length per field of view (FOV) and number of vascular junctions using ImageJ angiogenesis analyzer plug-in. n = 3 biological replicates. (C) Live cell images were taken every hour over a course of 24 h starting on day 3 upon supplementation of EMT-IC or control medium into co-cultures of A549 spheroids and MVNs. Representative enlarged close-ups of timeframes of pre-labelled A549 cells (red) and MVNs (green) overlayed with their respective phase contrast images taken at selected timepoints are displayed. Migrating and invading cancer cells are indicated by dashed circles. Time frames of whole spheroids can be viewed in Suppl. Figs. 4 and 5, whole 24h timeframes of close-ups can be viewed in Suppl. Figs. 6 and 7, while corresponding movies can be viewed in Suppl. Fig. 8 n = 11 biological replicates.

To visualize intravasation events in microfluidic devices, A549 spheroids were co-seeded with HUVECs into hydrogel center channels of microfluidic devices, and MVNs were allowed to form for the first 3 days of culture (Fig. 3H). Subsequently, the medium was changed to EMT-IC or unconditioned EGM-2 (control), and cultures were incubated for another 24 h. Evident from the phase contrast images, MVNs formed in close proximity around A549 spheroids, and were equally distributed throughout the chip, while tumor masses remained intact (Fig. 4A). Irrespective of the medium chosen, MVNs exhibited comparable stability on day 4 (Fig. 4B).

### 3.4 EMT-IC facilitated cancer cell intravasation into MVNs

Live cell imaging of fluorescently labelled A549 spheroids and MVNs was observed over 24 h to trace events of intravasation into MVNs (Fig. 4C, Suppl. Figs. 4 and 5). As cells were labelled with a non-permanent membrane dye, it did not outline the whole cell body after several days of culture. Hence, fluorescent labels were used as indicators of cell identity, while phase contrast (PhC) images were necessary to confidently track the movement of cells. Live cell imaging was performed at 10x to track the movement of all cells from a spheroid (Suppl. Figs. 4 and 5), while time-frames of observed intravasation events were presented in enlarged close-ups (Fig. 4C, Suppl. Figs. 6 and 7) and movies (Suppl. Fig. 8A–D). Overlays of fluorescent images and phase contrast allowed to observe cancer cells moving away from cancer spheroid, towards and along vessel walls. At sites of intravasation, cancer cells extended membrane protrusions through the microvascular wall, then maneuvered their bodies through the openings into MVNs (Fig. 4C, dashed circles). This was observed in control as well as EMT-IC conditions (Fig. 4C, Suppl. Figs. 4–8).

**Fig. 5:**
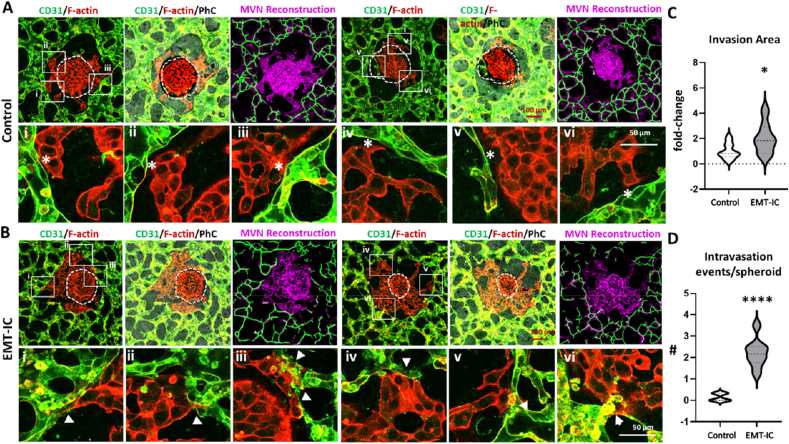
**EMT-IC (TGF-β1 with Mϕ-CM) facilitated A549 cancer cell intravasation into MVNs.** Day 4 co-cultures of A549 spheroids and MVNs, optionally exposed to EMT-IC for the last 24 h, were co-stained for CD31 and F-actin. (A) and (B) 20× magnification confocal microscopy images of co-stained CD31 and F-actin in control co-cultures and under EMT-IC exposure, respectively. Dashed white lines indicate initial A549 tumor masses. White rectangular frames indicate areas of close-ups, which are displayed in the rows below. Migratory non-intravasating A549 cancer cells are indicated by white asterisks, while intravasating cancer cells are indicated by arrow heads. To view intravasation events, MVNs were reconstructed and visualized using Qiber3D [54]. (C) Invasion area of A549 spheroids in microfluidic devices after 24 h of incubation. The degree of invasion of A549 cells into their surrounding environment was defined as the total area of invasion divided by initial area of the spheroid and normalized to control. (D) Manual semi-quantification of intravasation events per spheroid. ∗, p < 0.05, ∗∗∗∗, p < 0.0001. Control = 19 spheroids; EMT-IC = 21 spheroids collected from 3 biological replicates.

**Fig. 6:**
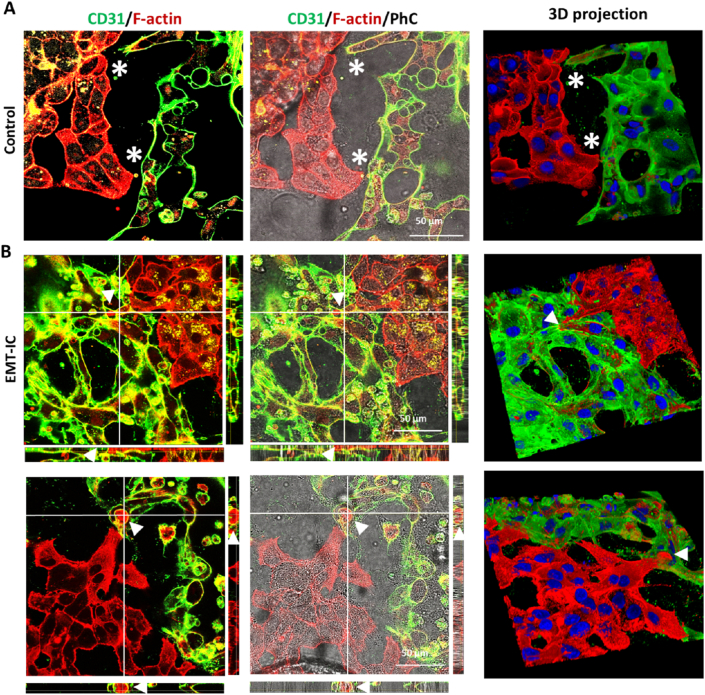
**High resolution confocal microscopy images visualizing intravasation events under EMT-IC**. High resolution confocal microscopy images (63x) including z-stack images displayed as orthogonal view and 3D projection of (A) Control and (B) EMT-IC conditions. Locations of cross-sections are indicated by white lines and orthogonal cross-sections are displayed on the sides. n = 3 biological replicates.

To visualize intravasation events in more detail, after 24h of exposure to control medium or EMT-IC, co-cultures of A549 spheroids and MVNs were fixed and co-stained for endothelial marker CD31 and F-actin. A549 exhibited a stronger fluorescent intensity for F-actin staining, enabling them to be distinguished from the CD31-positive, F-actin-weaker stained MVNs. As expected, cancer cells migrated out of A549 spheroids (initial tumor masses are indicated as white dashed lines) under both conditions (Fig. 5 A, B). Nonetheless, semi-quantification of invaded area by migrating cancer cells demonstrated that cancer cells exposed to EMT-IC exhibited an increased invasion potential in fibrin hydrogels within microfluidic devices (Fig. 5C). This was comparable to observations made in collagen I hydrogels previously (Fig. 2G). In control conditions, close-ups of confocal microscopy images (Fig. 5 A, B white solid rectangular frames) revealed that although cancer cells were in proximity of microvessels, commonly clear borders remained visible (Fig. 5A, white asterisks), suggesting that no intravasation events took place. In contrast, under EMT-IC, migratory cancer cells extended membrane protrusions into microvascular structures (Fig. 5B, arrowheads), thus initiating intravasation. Manual quantification of intravasation events, as defined in Fig. 5B, indicated an average of two intravasation events per spheroid when exposed to EMT-IC. In contrast, intravasation events were rare (<1 intravasation event/spheroid) under control conditions (Fig. 5D). This was further confirmed by high-resolution confocal microscopy, where clear separations between invading cancer cells and MVNs were mainly observed under control conditions (Fig. 6A, white asterisks). Under EMT-IC, cancer cell membrane protrusions invaded microvascular structures, followed by maneuvering their cell bodies into the MVN lumen (Fig. 6B, arrowheads). The latter was particular evident, as cancer cell bodies appear surrounded by CD31 stained lumen in orthogonal cross-sections and in 3D projections (Fig. 6B, second row).

**Fig. 7:**
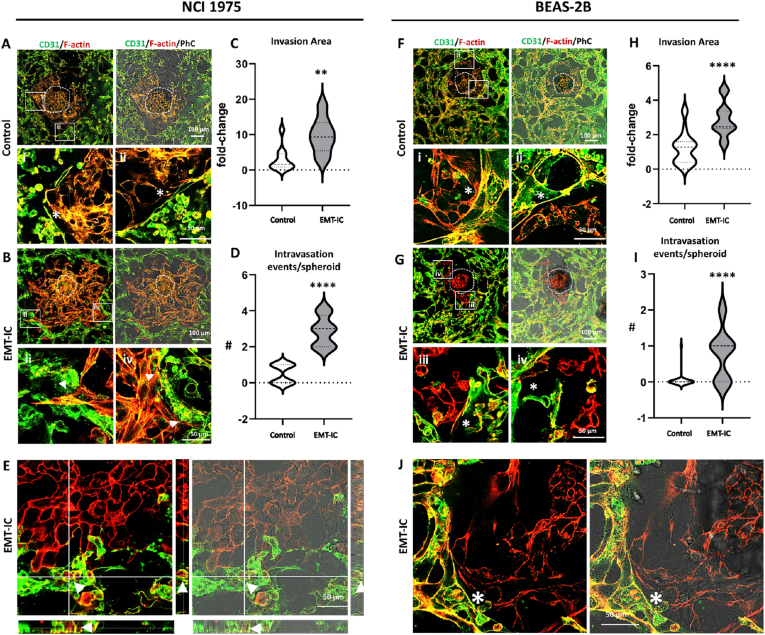
**EMT-IC (TGF-β1 with Mϕ-CM) facilitated NCI-H1975 and BEAS-2B cancer cells intravasation into MVNs to various degrees.** NCI-H1975 or BEAS-2B spheroids were co-cultured with MVNs in microfluidic devices and optionally supplemented with EMT-IC on day 3 for 24. On day 4 cells were stained for CD31 and F-actin. Figures A–E depict NCI-H1975 and figures F–J BEAS-2B cultures, respectively. (A) and (F) 20x confocal microscopy images of control conditions. (B) and (G) 20x confocal microscopy images of EMT-IC-treated conditions. White dashed lines mark the original tumor boundaries and white boxes highlight regions shown in the close-up panels below. Migrating but non-intravasating cancer cells are marked with asterisks, while arrowheads indicate intravasating cells. (C) and (H) Invasion area of cancer spheroids in microfluidic devices after 24 h of incubation. The degree of invasion of cancer cells into their surrounding environment was defined as the total area of invasion divided by initial area of the spheroid and was normalized to control. (D) and (I) Manual semi-quantification of intravasation events per spheroid. (E) and (J) High resolution confocal microscopy images (63x) including z-stack images displayed as orthogonal view of EMT-IC treated NCI-H1975 or BEAS-2B co-cultures with MVNs, respectively. Locations of cross-sections are indicated by white lines and orthogonal cross-sections are displayed on the sides. ∗∗, p < 0.01 and ∗∗∗∗, p < 0.0001. NCI-H1975: Control = 10 spheroids; EMT-IC = 8 spheroids collected from 3 biological replicates. BEAS-2B: Control = 22 spheroids; EMT-IC = 17 spheroids collected from 3 biological replicates.

In order to vailidate the universal applicability of the established lung cancer intravasation-on-a-chip model, two additional lung cancer cell lines: the non-metastatic BEAS-2B and the metastatic NCI-H1975 were introduced into the model (Fig. 7). Even under control conditions, the more aggressive metastatic NCI-H1975 cell line exhibited strong invasion potential, which could be extensively enhanced (10-fold) by EMT-IC (Fig. 7A–C). Congruently, NCI-H1975 cells were observed to frequently intravasate into the surrounding microvasculature under control conditions (0.5 events/spheroid), which could be substantially augmented by EMT-IC supplementation (3 events/spheroid) (Fig. 7D). Successful intravasation of NCI-H1975 cells under EMT-IC was confirmed in high resolution, where cancer cells were identified within the microvascular lumen (Fig. 7E).

In comparison, the response of BEAS-2B cells was substantially attenuated, as they invaded a qualitatively smaller area, which could only be slightly enhanced by EMT-IC (2-fold increase) (Fig. 7F–H). Furthermore, BEAS-2B cells did not exhibit any significant intravasation potential under control conditions, which could only be slightly elevated by EMT-IC, thereby maintaining baseline intravasation levels of less than 1 event/spheroid (Fig. 7I), corresponding to their non-metastatic nature. High resolution microscopy confirmed overall well defined boundaries between BEAS-2B cancer cells and MVNs, even under EMT-IC (Fig. 7J).

### 3.5 Machine learning (ML)-assisted quantification of intravasation events

In contrast to manual counting, which utilized 20x confocal images, live cell imaging of fluorescently pre-labelled A549 spheroids and MVNs over 24 h was used for ML-assisted quantification analysis. This was to ensure real-time observation of intravasation suitable for future applications. Any localization of red-labelled A549 cells within green-labelled MVNs was defined as an intravasation event. Live cell imaging confirmed augmented cancer cell shedding and migration, as well as an increased rate of A549 cells approaching the neighboring MVNs via extension of their membrane protrusions in the presence of EMT-IC (Fig. 4C, Suppl. Figs. 4–8).

**Fig. 8:**
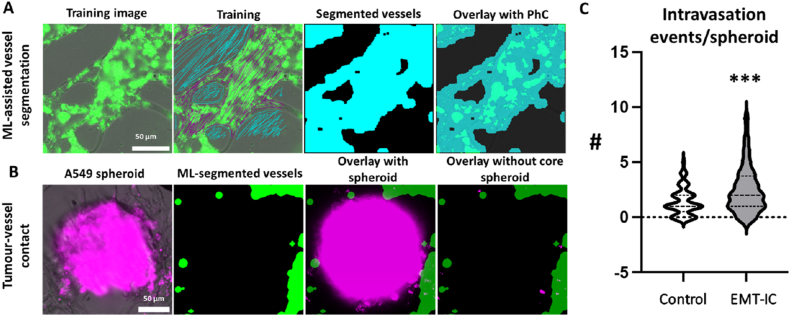
**Schematic overview of ML-assistedassessement of intravasation.** (A) vessel segmentation (Blue coloured lines during training: background; magenta-coloured lines during training: vessels; blue solid color: automatic segmented vessel recognition) and (B) tumor vessel contact quantification (Green solid colour: ML-segmented vessels; magenta colour: fluorescently labelled A549 cells; magenta colour of ‘overlay with spheroid’: binarized image of A549 spheroid). (C) ML-assisted automatic identification and quantification of intravasation events per spheroid. ∗∗∗, p < 0.001. Control = 72 spheroids; EMT-IC = 107 spheroids collected from 11 biological replicates.

An image analysis workflow was developed to segment vessels and cancer cells and analyze their co-localization to quantify intravasation events in an unbiased manner. Using the TWS plugin [59] (Suppl. Fig. 9), we reliably segmented the MVNs around the cancer spheroids. Combined with threshold-based segmentation of the cancer spheroids, we counted fluorescently labelled cancer particles within the segmented vessel area as a proxy for intravasation events (Fig. 8A and B). Our automated method showed similar trends to manual counting (Fig. 5D, Suppl. Fig. 10). We found, on average, three intravasation events per spheroid when exposed to EMT-inducing conditions (EMT-IC) and one intravasation event per spheroid under control conditions (Fig. 8C). Interestingly, the total number of events per group was slightly increased with the automated workflow.

### 3.6 The effect of EMT-IC on the stability and integrity of MVNs in co-culture with A549 spheroids

**Fig. 9:**
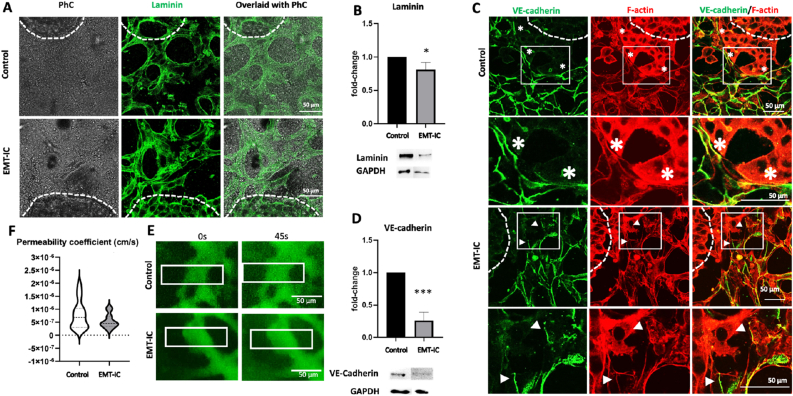
**The effect of EMT-IC (TGF-β1 with Mϕ-CM) on the stability and integrity of MVNs in co-culture with A549 spheroids.** MVNs and A549 spheroids were co-cultured in microfluidic devices and immunostained for laminin and VE-cadherin. (A) Confocal microscopy images of immunostained laminin, optionally overlayed with phase contrast (PhC) images. Borders of initial tumor masses are indicated by white dashed lines. (B) Western blot and densitometric band analysis of laminin normalized to their respective GAPDH levels of samples collected on day 4. Protein levels are displayed as fold-change as compared to control. (C) Confocal microscopy images of co-stained VE-cadherin and F-actin. Borders of initial tumor masses are indicated by white dashed lines. Areas of close-ups are indicated by solid line rectangles, and close-ups are displayed in the row below. Close interaction between cancer cells and MVNs with clear borders is indicated by white asterisks. Close interaction between cancer cells and MVNs with non-obvious borders is indicated by white arrowheads. (D) Western blot and densitometric band analysis of VE-cadherin normalized to their respective GAPDH levels of samples collected on day 4. Protein levels are displayed as fold-change compared to control. ∗, p < 0.05; ∗∗∗, p < 0.001, n = 3 biological replicates. (E) Representative frames from live cell imaging showing the perfusion of MVN from one medium channel to the other on day 4. (F) Permeability coefficient of MVNs for FITC-PVP (40 kDa) perfused under control and EMT-IC conditions on day 4. Control: n = 16, EMT-IC: n = 17, collected from 3 biological replicates.

Immunostaining of MVNs and A549 spheroids on day 4 revealed that both MVNs and tumor masses (identified as dense cell masses in phase contrast (PhC) images) synthesized laminin (Fig. 9A), while semi-quantitative evaluation of Western blot analysis of co-cultures revealed a slight decrease in overall laminin protein levels under EMT-IC (Fig. 9B). As expected, immunocytochemistry for VE-cadherin revealed that this cell adhesion molecule was restricted to MVNs, while F-actin was labelled in all cells in the co-culture. Nonetheless, A549 exhibited stronger fluorescent intensity for F-actin, enabling them to be distinguished from MVNs. This allowed a clear visualization of cancer cells migrating out of the initial tumor masses and towards MVNs (Fig. 9C). It is noteworthy that, in unconditioned medium, clear boundaries between migrating tumor cells and microvascular structures could be observed (Fig. 9C, asterisks), while under EMT-IC, those boundaries were not apparent at sites of cancer cell-MVN interaction. Indeed, VE-cadherin appeared downregulated at these specific locations (Fig. 9C, arrowheads). Semi-qantification of Western blot results for VE-cadherin demonstrated a more than 50 % decrease in culture with EMT-IC (Fig. 9D).

As vascular barrier functions, characterized by low vascular permeability, rely on tightly connected cell-cell junctions and a well-structured basement membrane, besides others [73], vascular wall permeability was next assessed. For this HUVECs were seeded into the medium channels of the cancer intravasation-on-a-chip on day 2 of culture to form an additional continuous endothelial monolayer at the media-hydrogel interface. By day 4, these endothelial cells had anastomosed with the MVN in the center channel, creating vascular openings that connected to the medium channels. To assess perfusion capability, FITC-PVP (40 kDa) was introduced together with EMT-IC into one of the medium channels, and live fluorescence microscopy demonstrated that MVNs were perfusable from the medium channels under both conditions (Fig. 9E). Next, vascular permeability was evaluated by measuring the diffusive flux of solutes across the vessel wall [53]. Specifically, the transport of FITC-PVP across the microvascular wall was quantified by tracking changes in fluorescence intensity within a defined perivascular (hydrogel) region over time (imaging every 15 s for 15 min). Assuming the microvessels formed circular tubular structures with diameters below 50 μm, the permeability coefficient was calculated using established methods [53].

Under initial exposure to EMT-IC, the MVNs exhibited a permeability coefficient of 7.79 ± 2.7 × 10^−7^ cm/s, which is comparable to that in control, which has a permeability coefficient of 5.41 ± 5.37 × 10^−7^ cm/s (Fig. 9F). Measurements of vascular permeability after 24h of exposure to EMT-IC were not possible, as vessel openings to media channels had closed at this point of the experiment under EMT-IC, while MVNs under control conditions remained perfusable.

## 4 Discussion

We have successfully established a microphysiological *in vitro* model of EMT-driven lung cancer intravasation coupled with ML-assisted image processing, enabling automated and unbiased quantification of intravasation events. This platform technology enables the visualization and investigation of underlying biological processes in high spatio-temporal resolution and its potential utilization as a drug screening tool. The universal applicability and sensitivity of the lung cancer intravasation-on-a-chip was demonstrated by the functional incorporation of various metastatic and non-metastatic lung cancer cell lines. Importantly, the baseline metastatic potential of cancer cell lines was clearly reflected in the measurements of invasion and intravasation potential in the devices, suggesting that the established model allows the prediction of cell specific metastatic potential and responsiveness to EMT induction, thus paving the way for testing of patient-specific samples.

A key advantage of our model is its accelerated intravasation timeline, as live cell imaging data confirmed that intravasation happens frequently within the first hours upon EMT induction. The 24-h endpoint thus provides a snapshot of actively intravasating cells, while early-intravasated cells are less likely to have migrated beyond the imaging field through MVNs. Importantly, the established system maintains excellent reproducibility within these first 24 h. This compressed timeline represents a significant advantage over both alternative *in vitro* models [[31], [34], [74], [75]] and *in vivo* [[76], [77], [78]] systems, particularly for high-throughput applications.

Moreover, a novel EMT induction cocktail based on the synergistic effects of macrophage conditioned medium and TGF-β was established, inducing a robust migratory and invasive behaviour in lung cancer cells, outperforming standard EMT induction methods. As both M1 and M2 polarized macrophages were implicated in facilitating EMT in cancer cells [79], we opted for unpolarized macrophages, which can secrete pro- and anti-inflammatory factors, and decided on moving forward with their conditioned medium, based on the abundance of EMT-inducers within. The M**ϕ**-CM contained a number of EMT inducers, including CHI3L1, IL-10, IL-6, IL-8, and TNF-α, which are known to play a role in cancer metastasis, migration, invasion, chemotaxis, and endothelial cell junction retraction [[80], [81], [82], [83], [84]]. The complexity of cytokines in our model more closely resembled the heterogeneous tumor microenvironment in advanced NSCLC [85], making it suitable for studying EMT-induced cancer intravasation mechanisms. We investigated successful EMT using various markers on protein and gene expression levels. Indeed, the EMT process is controlled by multiple transcription factors, including SNAI1, SNAI2, ZEB1, TWIST, CarB-box-binding factor, Mesenchyme Forkhead 1, and Kruppel-like factor [86]. These transcriptional regulators are modulated by intricate signaling networks within the tumor microenvironment, particularly through pathways involving TGF-β, Notch, and Wnt signaling [87]. Notably, the expression of these factors follows a temporal hierarchy during EMT progression: SNAI1 activation initiates the transition, while subsequent induction of SNAI2, ZEB1, and TWIST serves to sustain the resulting migratory phenotype [87]. Especially, ZEB1 and ZEB2 exhibited strongest upregulation in the EMT-IC condition, surpassing the effects of TGF-β1 alone. This is particularly significant as ZEB1 and 2 are linked to aggressive, stem-like phenotypes and immune evasion [88]. The synergy between M**ϕ**-CM and TGF-β1 on ZEB1 and 2 suggests that cancer cells adopted a more aggressive metastatic phenotype [89].

Besides marker expression we confirmed successful EMT on a functional level, where M**ϕ**-CM and TGF-β1 synergistically promoted migratory and invasive potential of cancer cells. Indeed, M**ϕ**-CM performed even better than TGF-β1, suggesting that the intrinsic biocomplexity of M**ϕ**-CM is advantageous for inducing EMT in A549 cancer cells. It is noteworthy that even though the conditioned medium of macrophages has been reported to induce EMT in colon cancer cells [90], this is the first study to investigate the synergistic effect of TGF-β1 and M**ϕ**-CM for EMT induction. Since the combination of TGF-β1 with M**ϕ**-CM appeared to act synergistically to facilitate EMT in A549 cells based on their marker expression, as well as functionality, this combination was chosen as EMT-IC for the microphysiological model.

Interestingly, the EMT-IC had a direct effect on MVNs, reducing their basement membrane and cell-cell junction proteins, respectively, thus potentially making them more permissive to intravasating cells [91], while not affecting their overall stability or vascular barrier functions to small solutes in the initial time frame investigated. Nonetheless, the effects of longer incubation times with EMT-IC were not tested.

The enhanced cancer cell invasion potential under EMT-IC, as observed in collagen type I hydrogels, was also reproduced in co-cultures with MVNs in fibrin hydrogels in microfluidic devices, suggesting that A549 cells, as well as other lung cancer cell lines underwent functional EMT in this co-culture set-up, albeit based on their metastatic predisposition [70]. Importantly, initiation of intravasation was mainly observed in co-cultures upon exposure to EMT-IC in metastatic lung cancer cell lines, where intravasation events were defined as cancer cells inserting membrane protrusions into microvascular structures. Besides the systemic effect of EMT-IC on vascular junctions, decrease in VE-cadherin was also specifically seen at sites of cancer cell entry. This suggests a direct communication of EM-transformed cancer cells and endothelial cells, resulting in permissive entry points in the microvessel walls [92]. With the established microphysiological model, these interactions can be visualized and studied under high spatio-temporal resolution, as well as investigated for potential drug targets for anti-cancer treatments.

Various microphysiological systems of cancer metastasis exist. They mainly focus on later stages of cancer metastasis, such as extravasation events [[7], [8], [9]]. It should be pointed out that physiologically representative models of cancer intravasation are worthwhile to focus on, as EMT-driven cancer intravasation serves as the rate-limiting step for circulating cancer cells [93]. Targeting the early stages of the metastasis cascade is thus likely more effective in preventing cancer cells from spreading [94].

Our model has several advantages over previously established cancer intravasation models. For many years, cancer research has been widely conducted in *in vitro* 2D cell culture and *in vivo* animal models. Although 2D monolayer cultures have been widely employed due to their high availability and reproducibility, these models are unable to model the etiology of the disease, to facilitate comprehensive cellular and environmental manipulation [[22], [32]]. Animal models also do not fully capture the complex biological processes that happen in the human body and have an inherently low resolution during real-time monitoring, limiting their read-out [[13], [15], [17]]. 3D *in vitro* microphysiological models provide a solution by bridging the gap between cell cultures and live tissues, enabling better control of the microenvironment while allowing the study of human physiology by using human cells [95]. In recent years, there has been significant advances in the development of various models of epithelial cancer intravasation, which aim to more closely resemble the primary and metastatic TME in an *in vivo*-like manner, particular on-a-chip models [[23], [24], [96], [97], [98], [99]]. However, most of these systems do not fully recapitulate the blood vessel anatomy as they focus on seeding endothelial on permeable membranes or hydrogel surfaces, thus still exhibiting properties of endothelial monolayers [32]. Endothelial monolayers, however, lack various functional features of proper microvasculature, such as physiologically representative barrier functions [100]. As barrier functions are one of the main obstacles cancer cells have to overcome to intravasate, the metastatic behavior of cancer cells cannot be properly modeled well with respect to the role of a functional microvasculature in these models. One cancer on-a-chip model utilized MVNs, which formed by vasculogenesis around cancer spheroids and exhibited improved barrier-properties [34]. Nonetheless, this study relied on the natural shedding of cancer cells from spheroids into MVNs and did not consider the process of EMT [34], thus rendering the study less physiologically relevant.

Our previously established approach to stabilize MVNs in microfluidic devices by utilization of macromolecular crowding (MMC) allowed the production of functional microvasculature-on-a-chip with hollow and circular lumina, apical-basal polarity and proper vascular barrier functions [48]. Importantly, the measured permeability coefficients of MVNs in the lung cancer intravasation-on-a-chip was closely aligned with *in vivo* measurements in rat venular vessels (1.37 ± 0.26 × 10^−7^ cm/s) [[40], [101], [102]], indicating that EMT-IC did not compromise vascular barrier functions and that the MVNs maintain physiological permeability levels.

Although MVNs formed spontaneously by vasculogenesis, their density was determined by initial cell seeding density and was reproducible across devices and biological repeats. These MVNs were then utilized to establish the lung cancer cell intravasation-on-a-chip model and thus investigate cancer intravasation into functional microvasculature. This approach is straightforward, thereby ensuring ease of use across different laboratory settings with high verifiability and reproducibility. Furthermore, in contrast to a previously reported intravasation-on-a-chip model [[23], [25], [103], [104], [105], [106]], our approach considered EMT as a major intravasation driver and was coupled with ML-assisted quantification. This model is, therefore novel, physiologically more representative and requires a shorter time to observe, visualize, and quantify intravasation events, enabling more efficient and less time-consuming experimentation and screening.

A general major limitation of *in vitro* cancer metastatic research is the time-consuming manual quantification of the rate of cancer intravasation. For example, a similar study used a ‘thin plate spline’ method to define tissue boundaries and compartment across multiple organ-on-a-chip models for quantifying tumor intravasation events [107]. Such mathematical modelling was required owing to variations that originate during organoid-on-a-chip manufacturing and experimental workflow [[108], [109], [110], [111]]. Automated image quantification utilizing ML-based approaches offers a solution, enabling unbiased analysis of large-scale data in various formats for tumor staging, cancer susceptibility, recurrence, and patient survival prediction [[96], [97]]. We developed an image analysis workflow using readily available, non-commercial and open-source tools to quantify intravasation events in a reliable and efficient manner. By defining intravasation events as the localization of fluorescently labelled cancer particles within segmented MVN areas, our automated method showed trends consistent with manual counting, underlining its reliability. Notably, our ML-assisted workflow detected, on average, one more intravasation event per sample than manual counting. This discrepancy may be due to the enhanced capabilities of ML-based analysis detecting intravasation events missed by the human eye. In addition, our image analysis workflow is universally applicable as it utilizes fluorescence signal overlap between tumor spheroids and endothelial cells to delineate intravasation events, similar to prior studies [[108], [109], [110], [111]]. Indeed, our quantification of intravasation events was similar to manual analyses and was robust throughout multiple biological runs.

ML-based tools are increasingly used in cancer research for staging, prognosis [112], extravasation [113], and lymph node metastasis [114]. Future advancements combining physiologically relevant *in vitro* models, such as ours, with 3D microscopy, 3D image analysis [54], and ML-assisted analysis will benefit drug development, early cancer detection and personalized treatment planning based on patient information [[115], [116]].

This platform technology has thus a significant impact by allowing the detailed study and visualization of intravasation processes over time. It enables the tracking of cells and provides high throughput capabilities for drug development. Currently, over 90 % of drugs fail in the clinical stage after years of costly development, primarily due to oversimplified *in vitro* models or non-translatable animal responses [117]. With this technology, drug candidates and therapeutic approaches can “fail fast-fail early”, thereby reducing costs and the reliance on animal experiments. This capability not only benefits the efficiency of drug development but also aligns with ethical responsibilities.

Future research directions in this field encompass utilizing lymphatic microvasculature [118], patient-derived cancer cells, incorporating additional components of the tumor microenvironment (such as extracellular matrix, cancer-associated fibroblasts, and tumor-associated macrophages), establishing diverse tests to further assess physiological representativeness, expanding the system to include other cancer types, and investigating the visualization of various biological and pathological processes, as well as drug responses.

The integration of ML with our EMT-driven lung cancer intravasation-on-a-chip model thus provides a physiologically relevant platform to mimic the initial process of cancer metastasis into microvasculature. This approach holds promise for improved drug development, personalized patient treatment plans and cancer progression prognosis.

## CRediT authorship contribution statement

**Christy Wing Tung Wong:** Writing – original draft, Visualization, Validation, Methodology, Investigation, Formal analysis, Data curation. **Joyce Zhi Xuen Lee:** Writing – review & editing, Investigation, Formal analysis, Data curation. **Anna Jaeschke:** Writing – review & editing, Software, Methodology, Formal analysis. **Sammi Sze Ying Ng:** Writing – review & editing, Investigation, Formal analysis. **Kwok Keung Lit:** Writing – review & editing, Methodology, Investigation. **Ho-Ying Wan:** Writing – review & editing, Methodology, Investigation. **Caroline Kniebs:** Writing – review & editing, Investigation. **Dai Fei Elmer Ker:** Writing – review & editing, Software, Methodology, Investigation, Formal analysis, Data curation. **Rocky S. Tuan:** Writing – review & editing, Supervision, Conceptualization. **Anna Blocki:** Writing – original draft, Supervision, Project administration, Investigation, Funding acquisition, Formal analysis, Conceptualization.

## Ethics approval and consent to participate

Not applicable.

## Funding

This work was supported by the research fund to the Center for Neuromusculoskeletal Restorative Medicine from Health@InnoHK program launched by 10.13039/501100003452Innovation and Technology Commission, the Government of the Hong Kong Special Administrative Region of the People's Republic of China (AB), by the Health and Medical Research Fund (08191066; AB) and by a direct grant (4054732, AB) from the Faculty of Medicine, CUHK. RST is supported by the Lee Quo Wei and Lee Yick Hoi Lun Professorship in Tissue Engineering and Regenerative Medicine. A.J. receives a Walter Benjamin postdoctoral fellowship from the Deutsche Forschungsgemeinschaft (DFG, German Research Foundation, Germany; 521343357).

## Declaration of competing interest

A provisional patent application concerning the cancer intravasation-on-a-chip model has been made.
