# Supplementary material for: Lung cancer intravasation-on-a-chip: Visualization and machine learning-assisted automatic quantification
Source: Bioact Mater. 2025 Jun 27;51:858–75. doi: 10.1016/j.bioactmat.2025.06.028 (PMC12269418; doi:10.1016/j.bioactmat.2025.06.028)
Supplement: Multimedia component 1 [file mmc1.docx]

**Supplementary figures for Lung Cancer Intravasation-on-a-chip: Visualization and Machine Learning-Assisted Automatic Quantification**


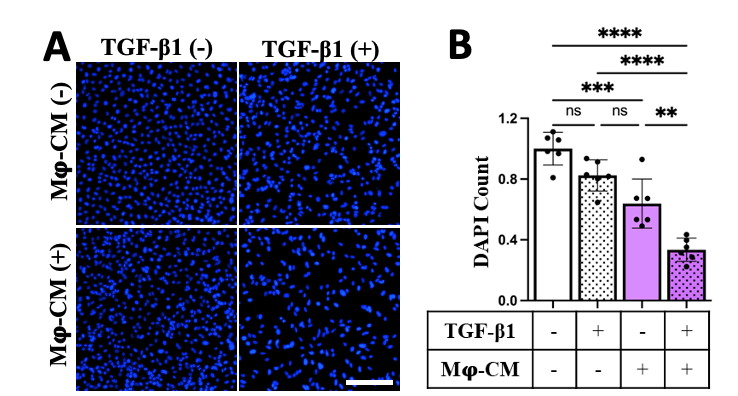


**Figure 1: Quantification of cell nuclei using DAPI staining.** A549 cells were incubated with TGF-β1, M𝛗-CM or combination of both for two days. (A) Representative images show DAPI-stained nuclei (blue). Nuclei were counted using particle analysis on ImageJ. (B) Bar graph represents the average number of nuclei per field of view, normalized to controls. **, p < 0.01; ***, p < 0.001; ****, p < 0.0001. n = 3 biological replicates.


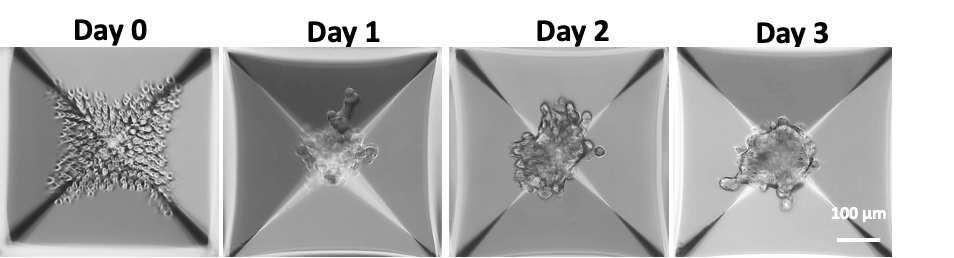


**Figure 2. Formation of A549 spheroids in microwells over time.** Representative micrographs depict the progression of A549 spheroid formation over a 3-day incubation period.


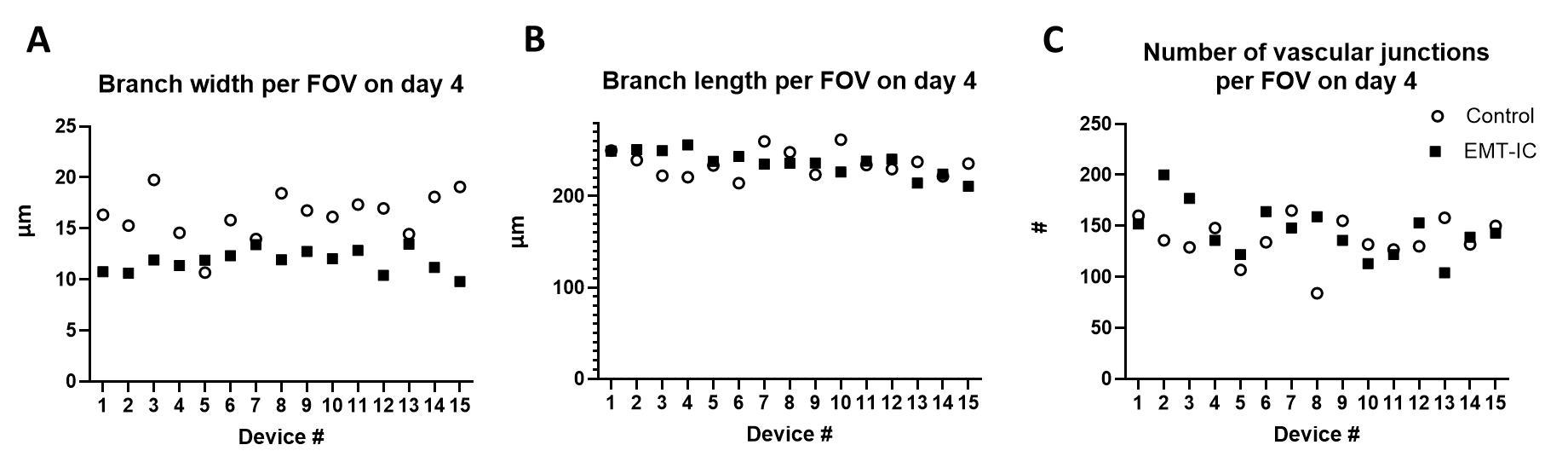


**Figure 3. EMT-IC (TGF-β1 & M𝛗-CM) did not adversely affect stability and integrity of MVNs.** Quantification of (A) Branch width, (B) tubular length and (C) number of junctions per field of view (FOV) using ImageJ angiogenesis analyzer plug-in.


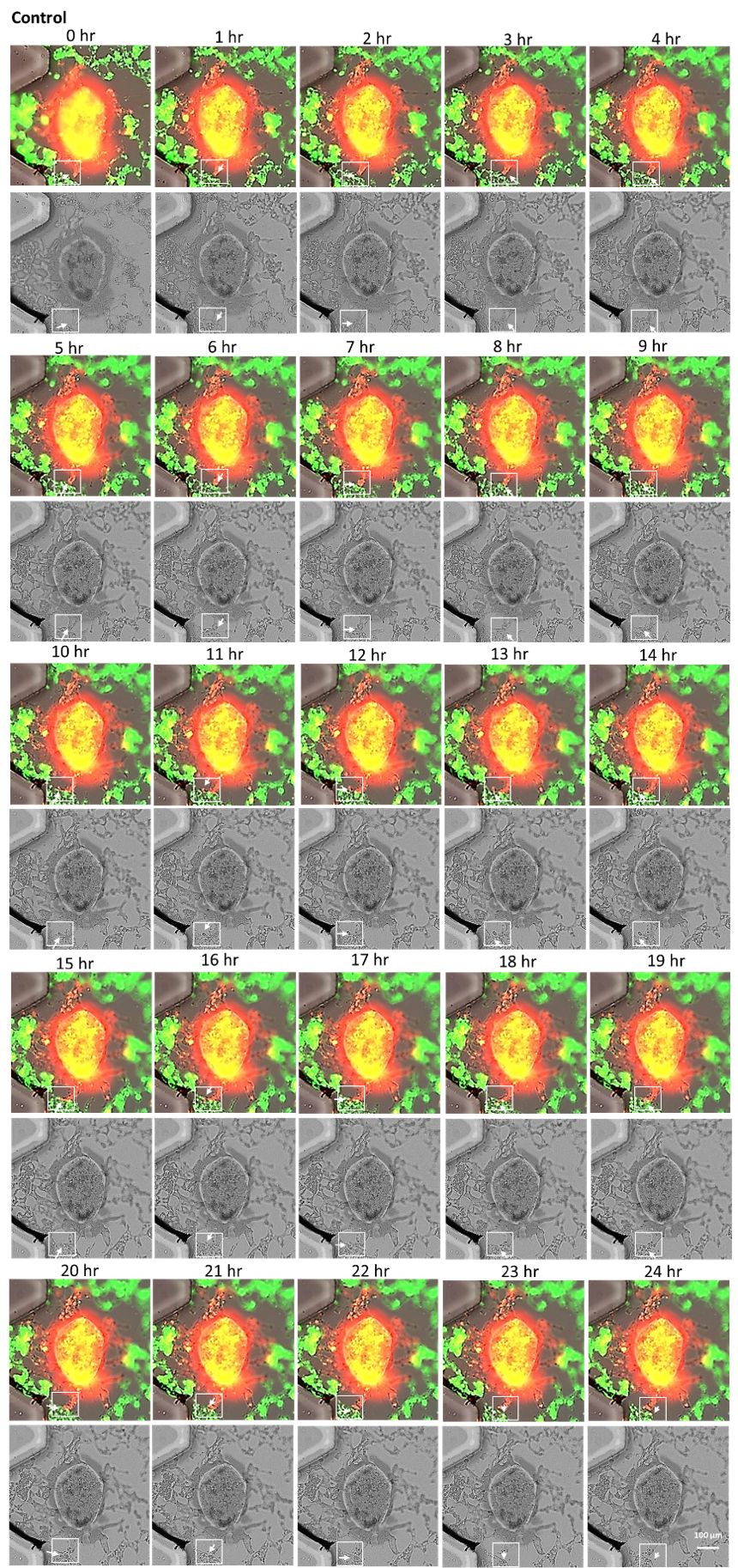


**Figure 4. Live-cell fluorescence imaging of A549 cancer cell spheroids co-cultured with HUVEC-derived MVNs over 24 hours.** Time-lapse live-cell imaging was performed hourly over a 24-hour period, starting on day 3 after supplementation with control medium into co-cultures of A549 spheroids and MVNs. Representative 10× magnification images display pre-labeled A549 cancer cells (red) and MVNs (green), along with corresponding phase contrast images. White arrows within boxed regions highlight migrating and invading cancer cells.


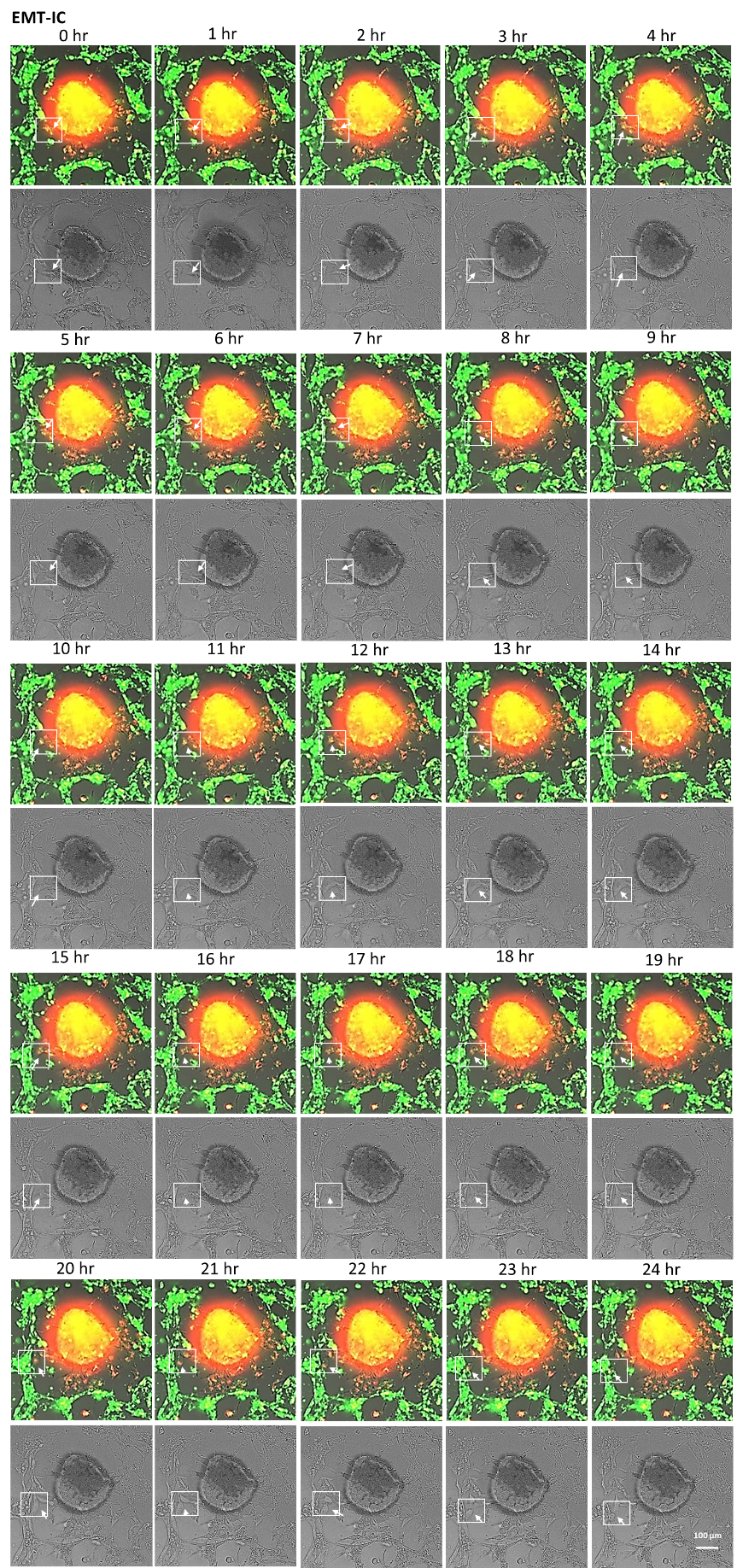


**Figure 5. Live-cell fluorescence imaging of EMT-IC-facilitated A549 cancer cell migration and intravasation into MVNs over 24 hours.** Time-lapse live-cell imaging was performed hourly over a 24-hour period, starting on day 3 after supplementation with EMT-IC into co-cultures of A549 spheroids and MVNs. Representative 10× magnification images display pre-labeled A549 cancer cells (red) and MVNs (green), along with corresponding phase contrast images. White arrows within boxed regions highlight migrating and invading cancer cells.


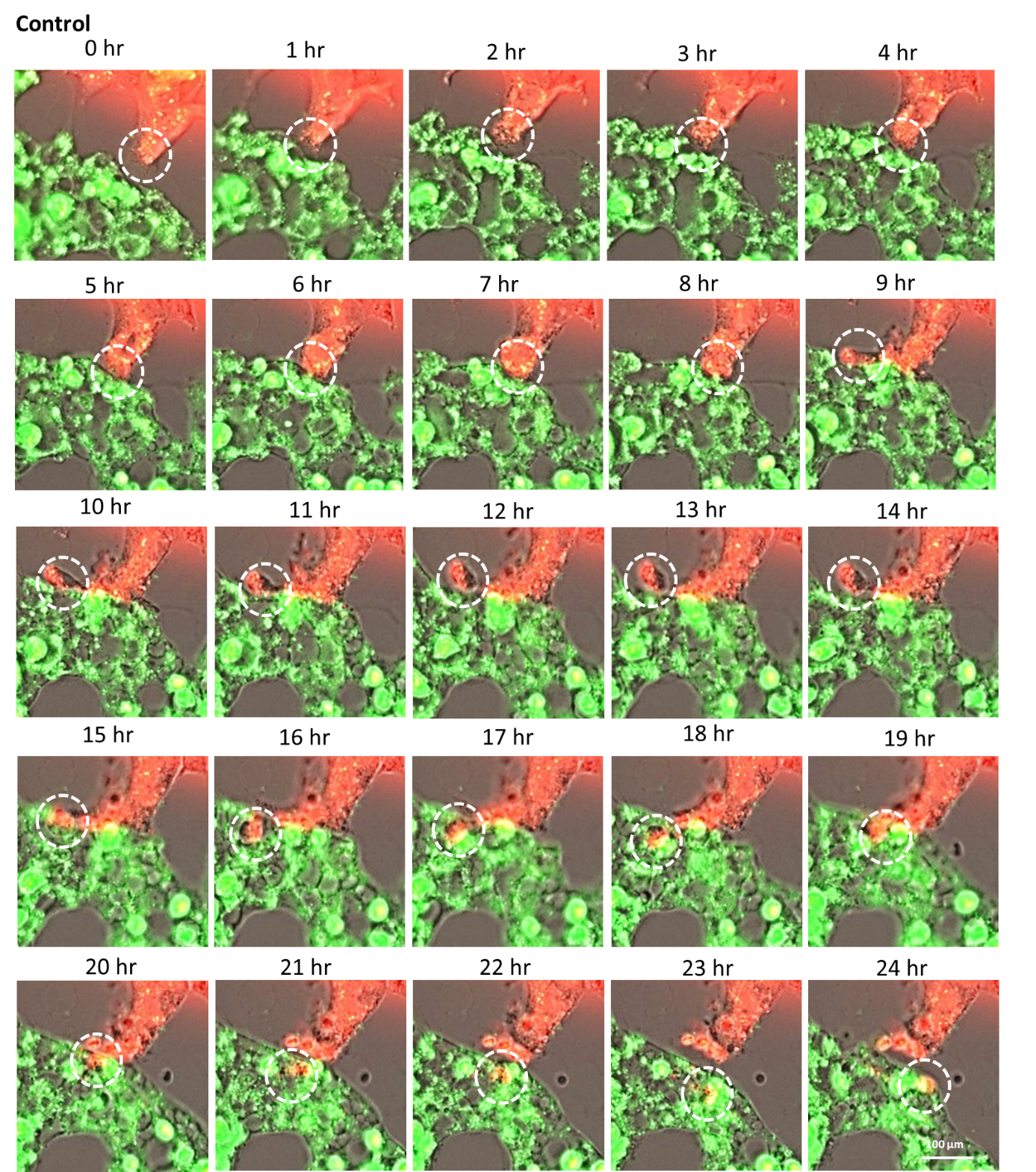


**Figure 6. Live-cell fluorescence imaging of A549 cancer cell spheroids co-cultured with HUVEC-derived MVNs over 24 hours.** Live cell images were taken every hour over a course of 24 hours starting on day 3 upon supplementation of control medium into co-cultures of A549 spheroids and MVNs. Representative enlarged close-ups of timeframes of pre-labelled A549 cells (red) and MVNs (green) overlayed with their respective phase contrast images taken every hour are displayed. Migrating and invading cancer cells are indicated by dashed circles.

**
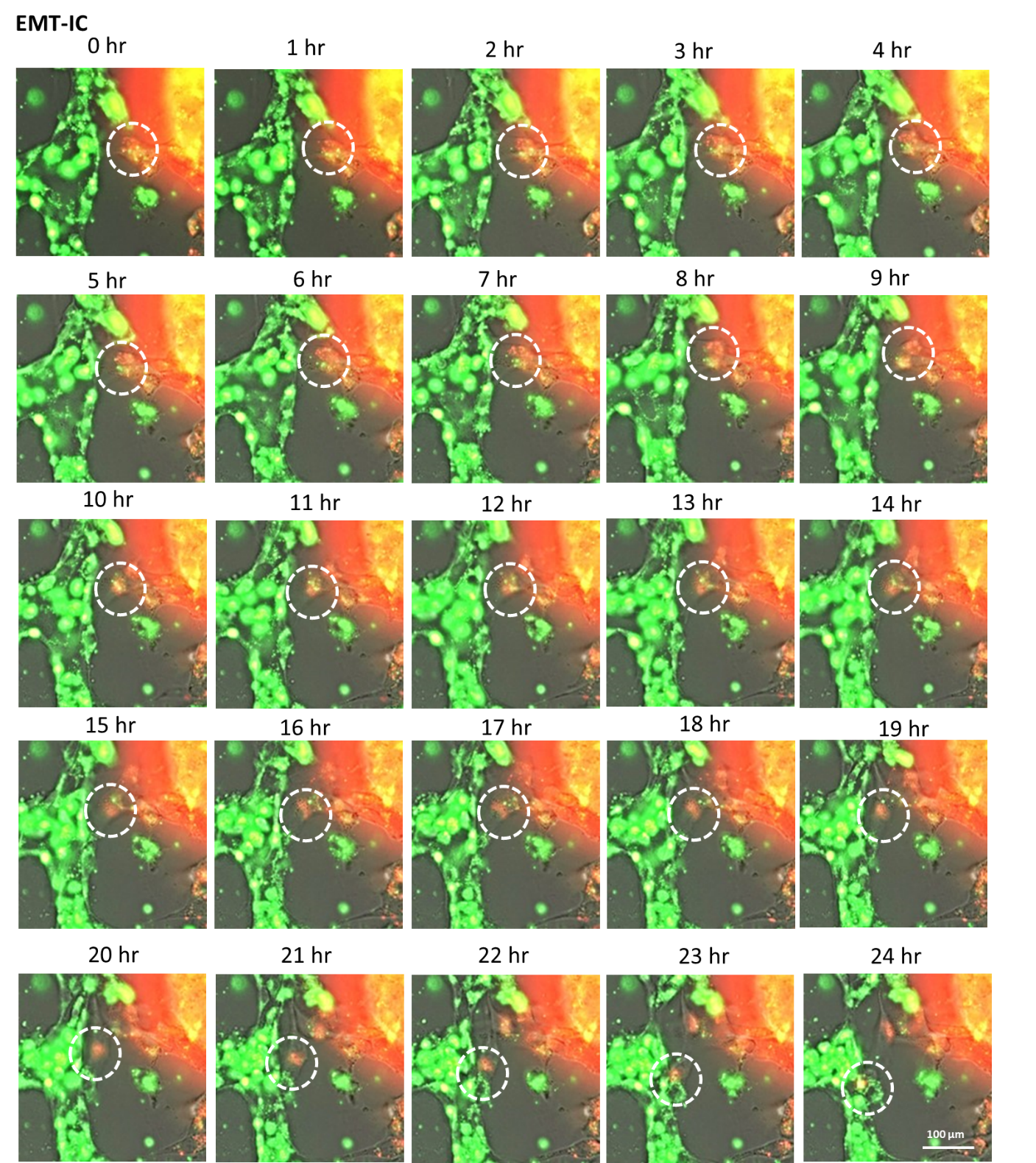
**

**Figure 7. Live-cell fluorescence imaging of A549 cancer cell spheroids co-cultured with HUVEC-derived MVNs over 24 hours.** Live cell images were taken every hour over a course of 24 hours starting on day 3 upon supplementation of EMT-IC into co-cultures of A549 spheroids and MVNs. Representative enlarged close-ups of timeframes of pre-labelled A549 cells (red) and MVNs (green) overlayed with their respective phase contrast images taken every hour are displayed. Migrating and invading cancer cells are indicated by dashed circles.

**
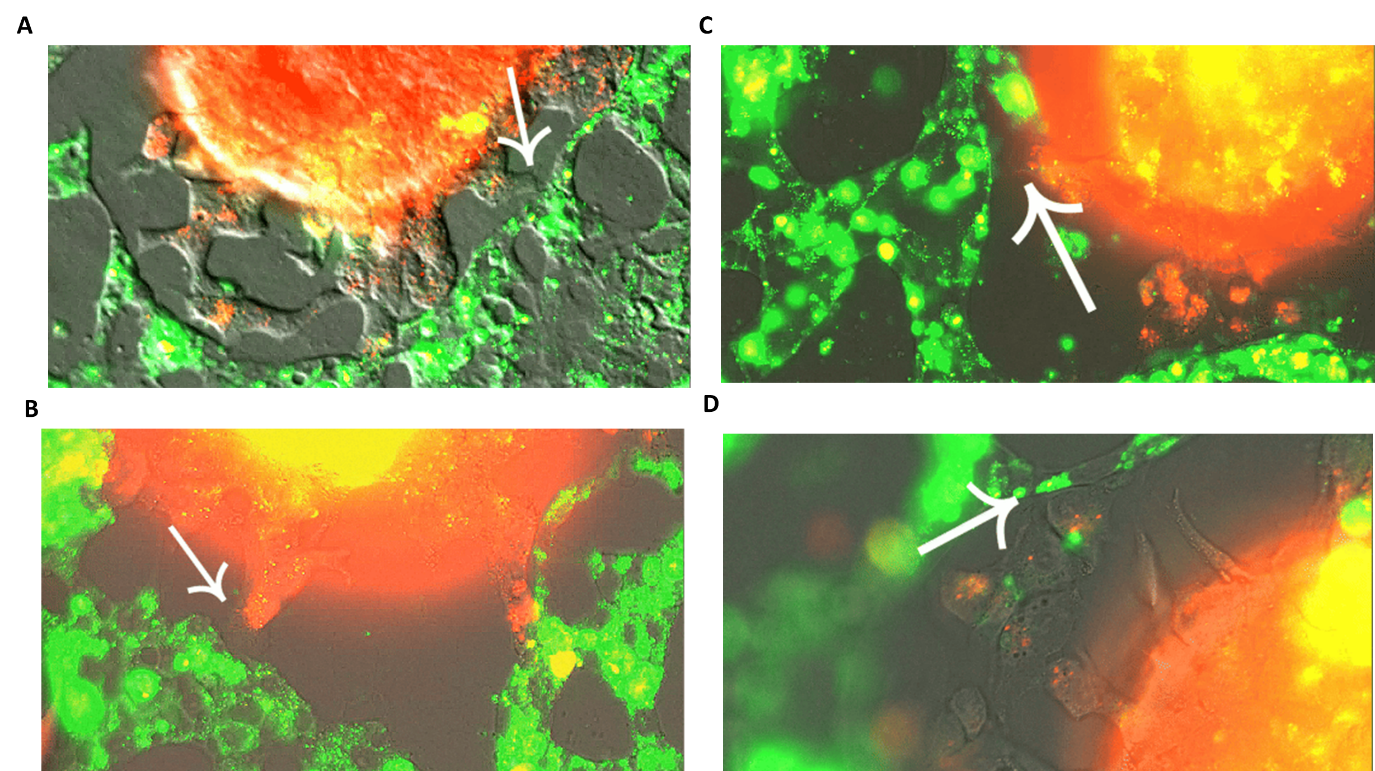
Figure 8. Time-lapse live-cell imaging of A549 cancer cell migration and intravasation over 24 hours.** Representative movies capture dynamic cell behaviors under different conditions over a 24-hour period. (A, B) Control condition and (C, D) EMT-IC condition, showing A549 cell migration and intravasation into MVNs. White arrows highlight migrating and invading cancer cells.


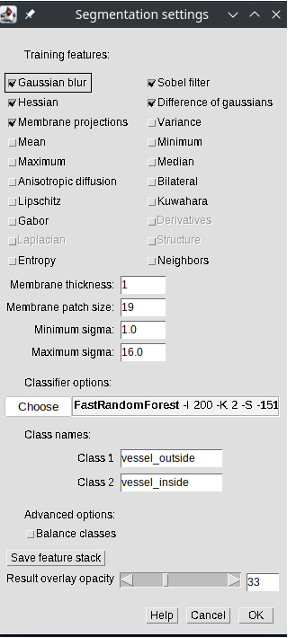


**Figure 9. Segmentation settings for image training using Trainable Weka Segmentation.** Overview of the parameters used for training images with Trainable Weka Segmentation, including selected training features, membrane thickness, and patch size. The Fast Random Forest classifier was utilized for segmentation. These settings were optimized to enhance accuracy and performance in distinguishing relevant structures.


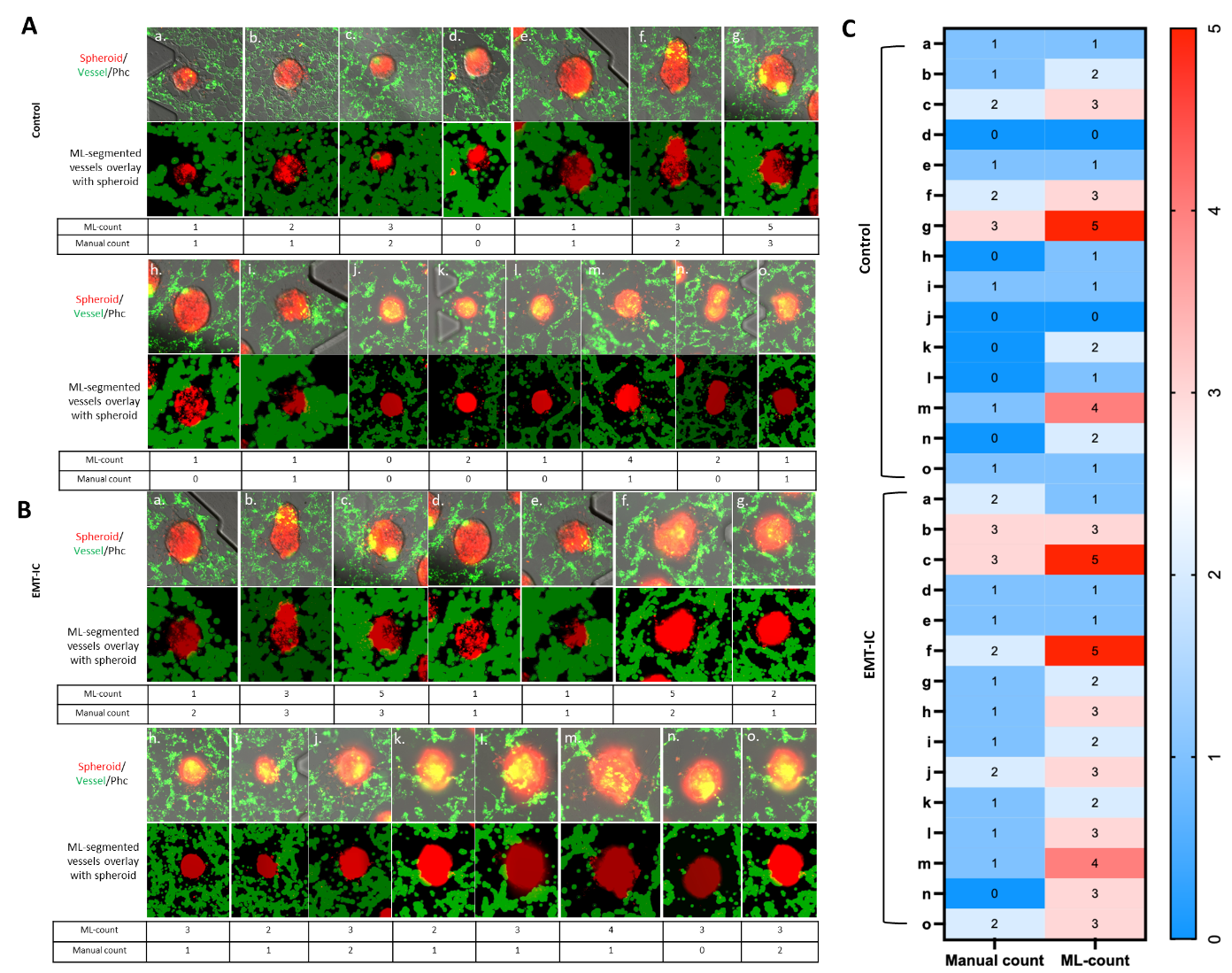


**Figure 10. Comparison of manual and ML-assisted quantification of intravasation events** in (A) Control and (B) EMT-IC conditions. (C) Heatmap showing intravasation events per spheroid for both methods. n=15 spheroids/condition.
