# Supplementary figures and images for: Lung cancer intravasation-on-a-chip: Visualization and machine learning-assisted automatic quantification
Source: Bioact Mater. 2025 Jun 27;51:858–75. doi: 10.1016/j.bioactmat.2025.06.028 (PMC12269418; doi:10.1016/j.bioactmat.2025.06.028)

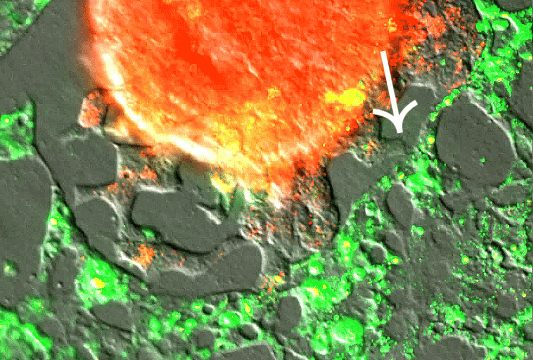

Supplement: Multimedia component 2 [file mmc2.gif]

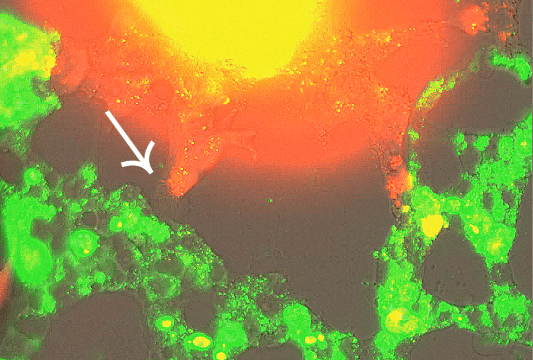

Supplement: Multimedia component 3 [file mmc3.gif]

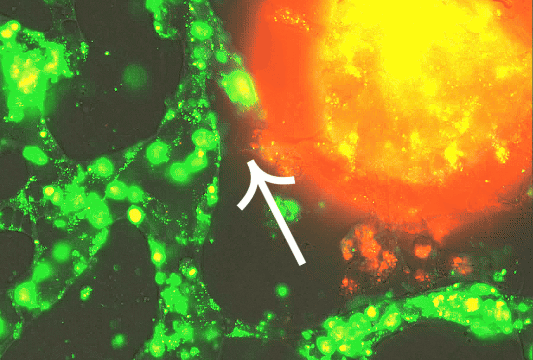

Supplement: Multimedia component 4 [file mmc4.gif]

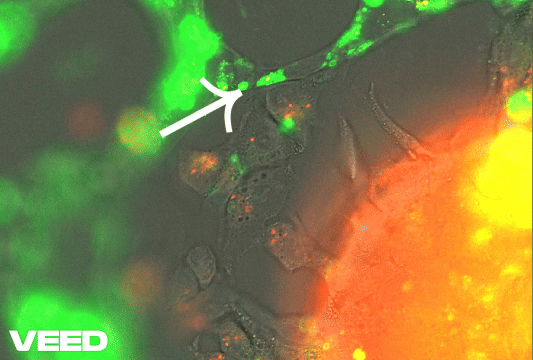

Supplement: Multimedia component 5 [file mmc5.gif]
